# Factors affecting interspecific differences in genetic divergence among populations of *Anolis* lizards in Cuba

**DOI:** 10.1186/s40851-018-0107-x

**Published:** 2018-08-09

**Authors:** Antonio Cádiz, Nobuaki Nagata, Luis M. Díaz, Yukari Suzuki-Ohno, Lázaro M. Echenique-Díaz, Hiroshi D. Akashi, Takashi Makino, Masakado Kawata

**Affiliations:** 1 Graduate School of Life Sciences, Tohoku University, Sendai, Japan; 2 Faculty of Biology, Havana University, Havana, Cuba; 3 National Museum of Natural History of Cuba, Havana, Cuba; 4 Miyagi University of Education, Aramaki-aza-Aoba, Aobaku, Sendai, Japan

**Keywords:** Genetic distance, *Anolis* lizards, Ecomorphology, Species age, Dispersal ability

## Abstract

**Background:**

Geographical patterns and degrees of genetic divergence among populations differ between species, reflecting relative potentials for speciation or cladogenesis and differing capacities for environmental adaptation. Identification of factors that contribute to genetic divergence among populations is important to the understanding of why some species exhibit greater interpopulation genetic divergence. In this study, we calculated the mean pairwise genetic distances among populations as species’ average genetic divergence by a phylogeny using nuclear and mitochondrial genes of 303 individuals from 33 Cuban *Anolis* species and estimated species ages by another phylogeny using nuclear and mitochondrial genes of 51 Cuban and 47 non-Cuban *Anolis* species. We identified factors that influence species’ differences in genetic divergence among 26 species of *Anolis* lizards from Cuba. Species ages, environmental heterogeneity within species ranges, and ecomorph types were considered as factors affecting average genetic divergences among populations.

**Results:**

The phylogenies presented in this study provide the most comprehensive sampling of Cuban *Anolis* species to date. The phylogeny showed more conservative evolution of *Anolis* ecomorphs within Cuba and identified twig anoles as a monophyletic group. Subsequent Phylogenetic Generalized Least Squares (PGLS) analyses showed that species age was positively correlated with species’ average genetic divergence among populations.

**Conclusion:**

Although previous studies have focused on factors affecting genetic divergence within species, the present study showed for the first time that species differences in genetic divergence could be largely affected by species age.

**Electronic supplementary material:**

The online version of this article (10.1186/s40851-018-0107-x) contains supplementary material, which is available to authorized users.

## Background

Genetic divergence among populations contributes to ecological divergence and subsequent speciation [1]. Additionally, greater genetic divergence with limited gene flows among populations reportedly promotes local adaptation and acquisition of potential to inhabit various environments within the range [2]. Thus, the identification of factors that contribute to genetic divergence among populations is useful to gaining a better understanding of why some species have greater interpopulation genetic divergence than others. Previous studies have mainly focused on factors that affect intraspecific differences in the genetic divergence among populations. For instance, it has been reported that geographically distant populations are likely to experience isolation owing to the distance [3]; thus, geographical barriers and distances may limit gene flow. Furthermore, genetic divergence among populations from different environments may be larger, owing to selection against individuals that move between the environments [4]. Hence, in addition to geographical isolation, isolation by environment is a major determinant of intraspecific genetic differentiation between populations, and the relative contributions of these factors and population histories have been investigated in several studies [5, 6].

However, average genetic divergence among populations also differs between species, and factors that affect interspecific differences in these genetic divergences among populations likely differ from those that affect intraspecific differences. Among potential predictors of average genetic divergence among populations, differences in species’ dispersal abilities or tendencies, which could be affected by several factors, influence gene flow and subsequently genetic divergence among populations. For instance, morphologically different species may differ in their dispersal ability [7, 8]. In addition, the effect of isolation based on environment might differ among species because individual dispersal tendencies with respect to preference of particular environments may have evolved depending on selective pressure and past environments experienced by the species.

Phylogenetic history [9] and species age [10] are crucial determinants of genetic divergence patterns and are responsible for current geographical species range. In particular, geographical distances over which gene flows occur increase with species ranges, and greater genetic differentiation has been shown among populations of various older species [9, 11–14]. However, few studies demonstrate the effects of species age on genetic divergence among populations within a species, and although Fujisawa, Vogler and Barraclough [15] correlated species occupancy (range size) with genetic variations in mitochondrial DNA sequences within species, correlations with species age were not identified.

*Anolis* lizards are among the most diverse vertebrate genera, and about 120 or more species have been identified in the Greater Antilles countries of Cuba, Hispaniola, Jamaica, and Puerto Rico [16]. *Anolis* lizards display a wide range of morphological and behavioral adaptations that are closely related to their most frequently used microhabitats. Hence, sets of species that share morphological, ecological, and behavioral traits are grouped into different ecological types, and are referred to as ecomorphs [17]. Cuba is the largest island in the Caribbean and has the highest diversity of *Anolis* lizards, with 64 known species [18]. Although classifications of ecomorphs vary between studies, Cuban ecomorphs have been defined as crown-giant, trunk-crown, trunk-ground, twig, and grass-bush. Although these ecomorphs have independently evolved on multiple Caribbean islands, some species show unique and independent evolution on islands that lacks ecomorphological classifications. Such anoles are considered to be unique and can be found within different clades among the Cuban *Anolis* [19].

Previous studies have indicated deep interpopulation genetic divergence in several Greater Antillean *Anolis* species [20–26], and deep mitochondrial divergence has also been demonstrated in species from the Lesser Antilles [27–31]. These studies show comparatively high levels of genetic divergence among populations of some species, leading to large differences in interpopulation genetic divergence among *Anolis* species. In a recent study, Wang, Glor and Losos [6] quantified relative influences of ecological (local environmental conditions) and geographical (distances between locations) factors on spatial genetic divergence in *Anolis* species from the Greater Antilles islands from Cuba, Hispaniola, Jamaica, and Puerto Rico. They showed that, although both geographical and ecological isolation significantly influence genetic divergence, the geographical isolation was substantially more predictive, suggesting that non-ecological factors play dominant roles in the evolution of spatial genetic divergences [6]. Wang et al. [6] also demonstrated that the effect of geographical distance on genetic divergence among populations was greater in some *Anolis* species than that in others; however, their study did not explore why some species have greater interpopulation genetic divergence than others.

In the present study, we identified determinates of interspecific (or interclade) differences in genetic divergence among populations of Cuban *Anolis* species. Specifically, we considered species age, environmental heterogeneity within species ranges, and ecomorph type as putative factors and tested the following hypotheses: (1) Older species are more likely to show greater genetic differentiation due to the greater time available for divergence. The effect of age on divergence can be detectable if the homogenizing effects of gene flows are not significantly large; (2) Species inhabiting more heterogeneous environments have evolved to adapt to different environments and thus have larger genetic divergence among populations than species inhabiting homogeneous environments; (3) Species that belong to different ecomorphs live in different microhabitats and show different moving abilities, which correlate with interspecific differences in genetic divergence.

Average genetic divergence is positively correlated with the average geographical distance among populations and this, in turn, is positively correlated with the size of the species’ range, which sometimes depends on the sampling locations between which the geographical distances are measured. Therefore, the average geographical distance can be used as a covariate of genetic distance and the above three factors could be examined by considering the effect of the average geographical distance for each species.

To test the hypotheses stated above, we used independent monophyletic clades as units of comparison. The members of populations within the clades potentially interbreed, so we examined factors affecting different degree of gene flows among populations within the clades. In addition, studied clades need to be independent and monophyletic so that the effects of population branching and separation from common ancestors can be assessed without gene flows from other clades. Using these units, we are able to evaluate whether genetic divergence within the clades was affected by emergent characteristics of the clade (e.g., environmental heterogeneity within species range, species age, etc.) or traits shared with all the populations of the species (e.g., morphology). In this study, in principle, we used species, but as shown below, some species form paraphyletic, not independent monophyletic clades. In such cases, we used independent monophyletic clades within the species as comparative units.

Herein, we focused on *Anolis* lizards in Cuba, since Cuba has the highest diversity of *Anolis* lizards among the Caribbean islands. We reconstructed a phylogeny for 33 Cuban *Anolis* species using nuclear and mitochondrial genes, and estimated pairwise genetic distances among populations for 26 of these. We also estimated divergence times using a reconstructed phylogenetic tree of 55 Cuban and 47 non-Cuban *Anolis* species. We then tested the explanatory value of the three factors outlined above for the observed genetic divergence among populations. Finally, to ensure that the effects of these factors were examined across a morphologically and ecologically diverse range of species, we analyzed a large number of species from Cuba.

## Methods

### Taxon sampling

**Fig. 1:**
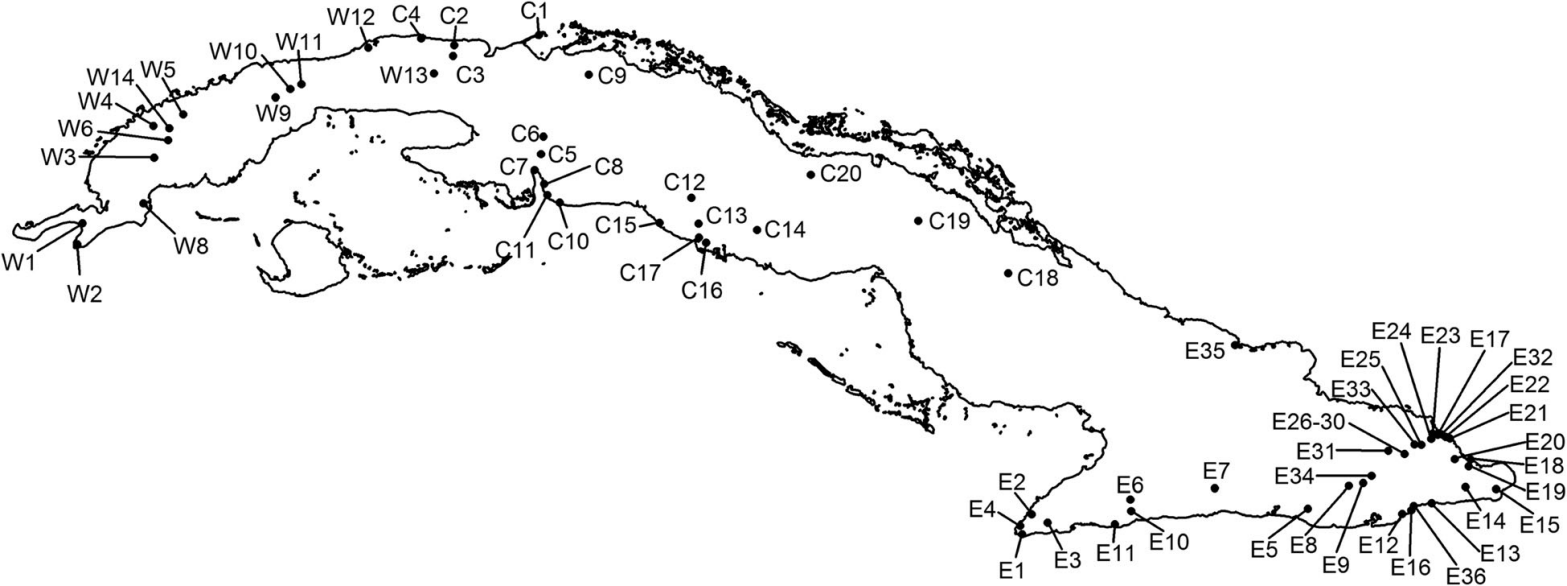
Map of Cuba showing sample locations; Additional file 1: Table S1 contains more detailed information regarding these locations

Field work was conducted during the wet seasons of 2008–2012 and was performed at 72 locations on the main island of the Cuban archipelago and on some smaller surrounding islands, such as Isla de la Juventud (Fig. 1). Sampling locations were selected to approximate known geographical ranges of each species based on previously published geographical distribution maps [32, 33]. Some individuals were preserved as voucher specimens and were deposited into the herpetological collection of the Graduate School of Life Sciences, Tohoku University, Japan. Tissue samples from other individuals were obtained by tail clipping and were stored in vials containing 90% ethanol. Locality data and GenBank accession numbers of samples from both this and a previous study [34] are summarized in Additional file 1: Table S1a. Ten Cuban species, *Anolis agueroi, A. birama*, *A. delafuentei*, *A. garridoi*, *A. incredulus*, *A. juangundlachi*, *A. pigmaequestris*, *A. terueli*, *A. toldo*, and *A. vescus,* were excluded from the present analyses. DNA sequences of four of these species (*A. delafuentei*, *A. incredulus*, *A. oporinus*, and *A. toldo*) were unavailable. The remaining species were not included in this study. Further, the inclusion of these species into the phylogeny did not alter the results. However, five species that had not been previously sequenced were added to the phylogeny (*A. anfiloquioi*, *A. fugitivus*, *A. spectrum*, *A. litoralis*, and *A. ruibali*). Numbers of samples and localities for each species are listed in Additional file 1: Table S1b.

### DNA extraction

Total DNA was extracted from tail or leg muscle tissues from each individual using standard phenol-chloroform methods. The mitochondrial gene encoding NADH dehydrogenase subunit 2 (*ND2*; 1036 bp) and the two nuclear genes encoding zinc finger protein 521 (*ZNF521*; 634 bp) and fibrosin-like 1 (*FBRSL1*; 488 bp) were amplified and sequenced from all individuals using the primer sequences and amplification conditions described by A Cádiz, N Nagata, M Katabuchi, LM Diaz, LM Echenique-Diaz, HD Akashi, T Makino and M Kawata [34].

### Phylogenetic analysis for intraspecific genetic distances

Two separate phylogenies were estimated using two different datasets. The first phylogeny was constructed for estimating intraspecific genetic distances as a proxy for genetic divergence among populations. The second was constructed for estimating divergence times and evaluating effects of species age on intraspecific genetic distances. The method for constructing the first phylogeny has been described in this section; the method for the second phylogeny has been described in the next section.

The first phylogeny was constructed using a dataset comprising 303 individuals belonging to 33 Cuban *Anolis* species. DNA sequences for species belonging to the trunk-ground ecomorph were obtained from a previous publication [34], and the phylogeny was constructed after selecting the localities on the main island of Cuba and excluding samples from the small surrounding islands. Two samples of Cuban *Leiocephalus* lizards (*L. onaneyi* and *L. raviceps*) were used as outgroups.

Genetic distances were estimated using a maximum likelihood (ML) tree that was generated from the combined data for the genes *ND2*, *ZNF521*, and *FBRSL1*. Tree topology was confirmed using Bayesian methods and prior to ML and Bayesian analyses, an appropriate model of sequence evolution and its parameters was determined using Kakusan 4.0 [35] The resulting GTR + Gamma+Invariant model [36] was applied to each of the regions based on the Akaike information criterion (AIC). ML analyses were performed using Treefinder [37] and robustness was validated using bootstrap analyses with 1000 replications. Bayesian analyses were performed to confirm ML topology using MrBayes version 3.1.2 [38]. Ten million generations of Markov Chain Monte Carlo (MCMC) were then performed with a sampling frequency of 1000. Subsequently, MCMC convergence was verified using Tracer 1.5 [39], and after discarding the first 2000 trees as burn-in, the remaining samples were used to estimate tree topology. Within-species pairwise genetic distances were calculated using the script distontree; http://www.fifthdimension.jp/products/distontree/. Generally, one or two (or in a small number of cases, three) samples per locality were used in the ML tree. However, when more than one sample from the same locality was analyzed, we calculated average genetic distances from all pairwise comparisons of samples from that location and from each of the other locations.

### Phylogenetic analysis for divergence times

The second phylogeny for estimating divergence times was constructed by using the mitochondrial *ND2* gene of Cuban and non-Cuban (from GenBank) *Anolis* species. Only mitochondrial *ND2* gene was used because the nuclear genes were not available for non-Cuban *Anolis* species. The dataset for the phylogeny comprised samples from single individuals of all species from each location. These were selected from all of the collected Cuban *Anolis* species (51 species, 221 samples in total), and included 15 species (*A. alfaroi*, *A. altitudinalis*, *A. anfiloquioi*, *A. baracoae*, *A. centralis*, *A. clivicola*, *A. guafe*, *A. guamuhaya*, *A. imias*, *A. litoralis*, *A. rejectus*, *A. ruibali*, *A. spectrum*, *A. vanidicus*, and *A. vermiculatus*) that were collected in one location, but were not included in the ML tree. In addition, 47 non-Cuban *Anolis* species that were representative of all Caribbean islands (Jamaica, Hispaniola, Puerto Rico, Bahamas, Caymans, and Lesser Antilles) and of the continent (Central and South America), were selected using the most complete phylogeny available [40] as a reference, and included 2–3 species from each of their main clades. Finally, gene sequences for three Cuban species were downloaded from GenBank (*A. macilentus*, *A. oporinus*, and *A. porcus*) and were included in this phylogeny. We also used GenBank sequences for *Basiliscus plumifrons* and *Polychrus acutirostris* as outgroup species. Accession numbers for non-Cuban species are presented in Additional file 1: Table S2.

Divergence times were estimated using the relaxed clock model in BEAST 1.6.2. In these analyses, the ML tree generated by Treefinder was used as an initial tree for BEAST, and one run with 10 million generations of MCMC was performed. After MCMC, convergence was confirmed using Tracer v1.5, and the first 2000 trees were discarded as burn-in. We set one calibration point at the root of all species as 95 Mya according to a study reported by Nicholson et al. [41]. Fossil evidence is largely lacking for anoles, making the calibration of trees difficult. However, the following analyses do not require accurate ages of divergence because we compared the relative age of divergence among species.

### Ecomorph classification

The ecomorph classification used here largely follows that of previous studies (e.g., [42, 43]), although we refined classifications for a group of 15 Cuban species that are currently considered unique anoles and therefore do not belong to any ecomorph class. We classified these Cuban unique anoles into unique-types 1, 2, 3, and 4 according to morphological, behavioral, and ecological characteristics, which may reflect dispersal abilities. Finally, we summarized the characteristics of each of these species groups (Additional file 1: Table S3), according to the studies by JB Losos [19] and L Rodríguez-Schettino [32].

### Species used for the analysis

The present analyses were performed with independent monophyletic species. However, because the geographical ranges of *A. ahli*, *A. barbatus*, *A. bartschi*, *A. confusus*, *A. cupeyalensis*, *A. fugitivus*, *A. quadriocelifer*, and *A. noblei* are highly restricted, we sampled fewer than three closely-located areas and excluded data for these species from analyses.

For some species, populations from different regions formed different paraphyletic groups in both ML and Bayesian trees. Among these, *A. allogus* populations in the eastern region belonged to a separate clade from those in the western region, which belonged to the same clade as *A. ahli* ([34]; the present study). Thus, the separate paraphyletic clades of *A. allogus* were considered different species. Moreover, the average genetic divergence between localities tended to become larger when genetic distances between populations of these two clades were included. Hence, *A. allogus* populations were divided into eastern and west-central clades. Similarly, clades of *A. allisoni* and *A. porcatus* exhibited interspecific hybridization ([20]; the present study), and were divided into eastern and central, and eastern and west-central clades, respectively, and groups of these populations were considered separate species in analyses of the factors affecting intraspecific genetic divergences.

Populations of *A. jubar* were not monophyletic [34], and were therefore removed from analyses. In addition, the *A. sagrei*, sample 07_8_sag_W3, was not included in analyses because it was nested within the clade of *A. bremeri*, suggesting hybridization at locality W3. A similar decision was made for sample 41_7_bre_W8 of *A. bremeri*, which appeared to hybridize with *A. sagrei* at locality W8.

A total of 26 species was included in analyses of the factors that affect intraspecific genetic divergence. These included three species of the crown-giant ecomorph (*A. equestris*, *A luteogularis*, and *A. smallwoodi*), three species of the grass-bush ecomorph (*A. alutaceus*, *A. cyanopleurus*, and *A. inexpectatus*), five species of trunk-crown anoles (*A. isolepis*, Eastern *A. allisoni*, Central *A. allisoni*, Eastern *A. porcatus*, and West-Central *A. porcatus*), seven species of the trunk-ground ecomorph (Eastern *A. allogus*, West-Central *A. allogus*, *A. bremeri*, *A. homolechis*, *A. mestrei*, *A. rubribarbus*, and *A. sagrei*), three species of the twig ecomorph (*A. alayoni*, *A. angusticeps*, and *A. guazuma*), three species of unique-type 1 (*A. argillaceus, A. pumilus*, and *A. loysianus*), and two species of unique-type 2 (*A. argenteolus* and *A. lucius*).

### Environments within species ranges

For each locality we extracted 19 bioclimatic variables from the WorldClim database (http://www.worldclim.org), as shown in Additional file 1: Table S4, and one vegetation variable (percent tree cover) based on satellite images of the entire globe from the MODIS sensor of Terra from the International Steering Committee for Global Mapping (https://globalmaps.github.io/ptc.html; Additional file 1: Table S4). Data of tree cover was used because the forest coverage could affect thermal environments that are important for distributions of anole species [34]. Data from WorldClim includes percent tree cover with 1-km resolution. Because anole samples were collected within a few kilometers of each sampling location, we averaged percent tree cover data with a 5-km resolution using ArcGIS ver. 10.0 so that the entire range of habitats was included for sampled populations. Values were then extracted for each variable at each location, and 20 environmental variables were normalized using principal components analysis (PCA). Subsequently, environmental dissimilarities (Euclidean distance) were calculated according to the variables on PCA axes 1–5 using R software (Additional file 1: Table S4).

Environmental heterogeneity between locations within species ranges was calculated and average environmental dissimilarity values were then calculated between species locations. These values were lower when the environments within the species ranges were homogeneous, and were increased with environmental differences between locations.

### Geographic distances

Pairwise geographic distances were estimated using Geographic Distance Matrix Generator v1.2.3 [44] with samples from localities on the main island of Cuba. For each species, average values of all pairwise genetic and geographic distances (km) between locations were regarded as average genetic distances and average geographic distances for species, respectively. Because different species have different ranges and distributions, the number of sampling locations and the geographic distances between the locations varied. However, sampling locations were selected for the best feasible coverage of known geographic ranges of each species, leading to greater numbers of sampling locations for species with larger ranges. In some cases, sampling locations were selected to optimize detectability of species, and may have been affected by population density. Thus, sampling locations were generally representative of each species and average genetic distances for each species therefore represented characteristic geographic distances among major localities of species.

Because sampling locations were selected to cover the largest possible proportion of the known geographic ranges for each species, average geographic distances were highly correlated with range sizes (*r* = 0.8509, *t* = 7.9375, *P* = 3.625 × 10^− 8^), which were determined using a previously published distribution map [32]. Therefore, we omitted sizes of species ranges from our analysis to avoid multicollinearity in generalized least squares analyses. Although average geographic distances for each species were considered characteristic of geographic distances and were used as a proxy for species range, these sometimes depended on sampling locations. Thus, although average geographic distances were not considered explanatory, they were included as a covariate that potentially affects genetic distances. Thus, we determined predictors of average genetic divergence with consideration of average geographic distances.

### Phylogenetic generalized least squares analyses

In phylogenetic generalized least squares (PGLS) analyses, we examine the effects of these factors on interspecific differences in genetic divergence within species, with consideration of the effects of phylogenetic constraints. In these analyses, average genetic distances among populations were entered as the response variable (estimated in section “Phylogenetic analysis for intraspecific genetic distances”), and average geographic distances between localities, ages of origin, average environmental dissimilarities and ecomorph types were included as explanatory variables. Average geographic distances between locations were included as a potential covariate of genetic distances. PGLS can consider the effect of phylogenetic constraints for the analysis and should include genetic distance matrix among species. For this purpose, the genetic distances among species were recorded and a phylogenetic tree was calculated from all individuals of the same species using the Maximum Composite Likelihood model in MEGA7 [45]. Because the genetic distance between species rather than individuals were used for removing phylogenetic constraints, we calculated genetic distances between species again using MEGA7. The resulting differences in composition biases among sequences were considered in previous evolutionary comparisons [46], and all positions containing gaps and missing data were eliminated. Subsequent PGLS analyses was conducted using the gls function in the “nmle” package of R (version 3.1.1), assuming a Brownian motion model of evolution.

Initially, PGLS analyses were performed using a full model that included all explanatory variables and potential interaction terms. Non-significant variables were then removed from the full model using a backward stepwise procedure with log-likelihood ratio tests, and the final model was constructed to include only significant variables and their interaction terms. Because PGLS could not be conducted using a model that only included the interaction terms, single variables that formed interaction terms were only included in the final model when they were significant in the full model. This final model was used to identify factors that are significantly associated with average genetic divergences within species.

## Results

### Phylogeny of *Anolis* species in Cuba

**Fig. 2:**
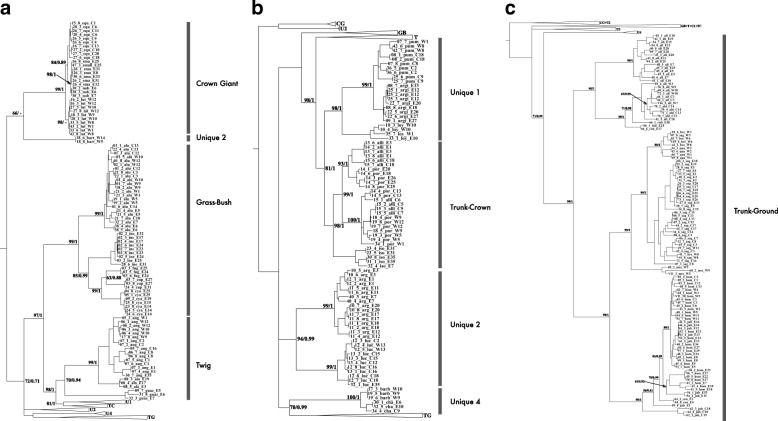
Maximum likelihood tree (50% majority consensus) based on three genes (2170 bp); 303 individuals from 33 Cuban *Anolis* species were included as the ingroup and two species from the genus *Leiocephalus* were included as the outgroup. Node supports are bootstrap percentages from maximum likelihood analyses; Bayesian posterior probabilities are shown only for the major clades. The figure has been split into parts A, B, and C, and some clades have been compressed to fit the full tree; CG, crown-giant; GB, grass-bush; T, twig; TC, trunk-crown; TG, trunk-ground; U1, unique-type 1; U2, unique-type 2; U4, unique-type 4; outgroup not shown

The present ML tree (Fig. 2) includes 303 individuals from 33 Cuban *Anolis* species and is more comprehensive than previous phylogenetic analyses of Cuban taxa. We incorporated a greater level of geographic sampling and a higher genetic resolution by including mitochondrial and nuclear markers. The topology of the ML tree was highly consistent with that generated using Bayesian inference (Additional file 2: Figure S1). Moreover, each of the present ecomorph classes formed monophyletic groups and similar results were obtained for groups of unique anoles, with the exception of the unique-type 2 species, which was polyphyletic. Accordingly, all twig anoles included in this tree (*A. angusticeps*, *A. guazuma*, and *A. alayoni*) were recovered as monophyletic species. These results were supported by the phylogenetic tree based only on the nuclear DNA tree (Additional file 2: Figure S2). Although previous studies classified these species as paraphyletic [40, 41], the present nodes were not well supported, warranting cautious conclusions of monophyly.

### Phylogeny of Cuban and non-Cuban *Anolis* species and estimates of relative divergence times

We generated a phylogenetic tree with estimated relative divergence times from BEAST (Additional file 2: Figure S3), using 101 in-group species (54 Cuban and 47 non-Cuban species) and two out-group species. In agreement with the above ML analyses, all Cuban ecomorphs were recovered as monophyletic species with the exception of one twig species (*A. guazuma*), which appeared to be polyphyletic and was closely related to the clade comprising unique-type 1 and trunk-crown anoles.

### Effects of average geographic distances, environmental heterogeneity, and species ages on interspecific differences in genetic divergence among populations

**Fig. 3:**
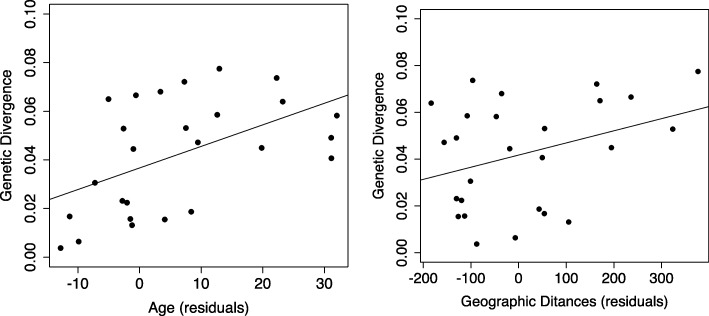
Relationship between average genetic divergences of species and species ages (**a**) and average geographic distances (**b**); residual regression values for species age and geographic variances vs. average genetic divergences were used

PGLS analyses showed that species age and geographic distance are significant predictors of average genetic distances between localities based on genetic distances that were calculated using nuclear and mitochondrial DNA (nDNA + mtDNA; Table 1a), or mtDNA only (Table 1b). Thus, older species tend to have larger genetic divergences (Fig. 3a) even when the significant effects of geographic distances are controlled. PGLS analyses with the full model also showed that environmental heterogeneity within species ranges and ecomorph types did not affect genetic distances among populations.

**Table 1:** Results of phylogenetic generalized least squares (PGLS) analyses for the final model using nuclear DNA (nDNA) and mitochondrial DNA (mtDNA) (a), and mtDNA only (b)

(a) nDNA+mtDNA				(b) mtDNA			
	d.f.	F-value	*P*-value		d.f.	F-value	*P*-value
(Intercept)	1	4.8154	0.0594		1	6.518581	0.0287
Species age	1	12.070760	0.0084	Age	1	8.767710	0.0143
Geographic distance	1	11.052356	0.0105	Geographic distance	1	19.783943	0.0012
Environmental heterogeneity	1	0.027673	0.8720	Ecomorph	6	0.891333	0.5358
Ecomorph	6	1.219130	0.3866	Geographic distance × species age	1	3.526034	0.0899
Geographic distance × species age	1	3.532252	0.0970	Geographic distance × ecomorph	6	2.114500	0.1412
Geographic distance × environmental heterogeneity	1	2.174493	.1785				
Geographic distance × ecomorph	6	2.102918	0.1631				

The effects of species age, ecomorph, and environmental heterogeneity within species ranges on average genetic divergence among populations of each species; geographic distances were included as a covariate. PGLS analyses were performed using a full model that included all explanatory variables and possible interaction terms. Non-significant variables were then removed from the full model using a backward stepwise procedure with log-likelihood ratio tests. The final analytical model included only significant variables and their interaction terms

## Discussion

### Phylogeny of Cuban anoles

The phylogenies presented in this study follow the most comprehensive sampling of Cuban *Anolis* species to date. In particular, the species *A. anfiloquioi*, *A. fugitivus*, *A. spectrum*, *A. litoralis*, and *A. ruibali* had not been sequenced previously and were added to the phylogeny. Furthermore, lizard samples of most species were collected from their entire known geographic ranges, and we improved the molecular resolution using multiple genetic markers. Previous phylogenetic analyses of most Cuban species [40, 41, 47] were performed using mtDNA only, although some studies on smaller groups of Cuban species have included nuclear markers [20, 34, 48]. In particular, the most recent and comprehensive phylogenetic analysis of Anoles [49] includes more Cuban species than were included in the present study, but the DNA sampling accomplished here is much more extensive than in the previous study. They included 61 species from Cuba, but they used only morphological data for some species and lacked molecular data for 13 of them: *A. toldo*, *A. litoralis*, *A. ruibali*, *A. terueli*, *A. juangundlachi*, *A. fujitivus*, *A. anfiloquioi*, *A. spectrum*, *A. vescus*, *A. pigmaequestris*, *A. delafuentei*, *A. incredulus*, and *A. birama*. Thus, we accomplished a better DNA sampling of *Anolis* of Cuba than Poe et al. [49] because we sequenced five species (*A. anfiloquioi*, *A. fujitivus*, *A. spectrum*, *A. litoralis*, and *A. ruibali*) for the first time and we sampled most species from several localities.

Although the present phylogenetic relationships are mostly consistent with those of previous studies [49, 50], Cuban twig anoles were identified as a monophyletic group in our analysis (Fig. 2), indicating a more parsimonious evolution of *Anolis* ecomorphs on the island of Cuba, albeit with poor node support for this monophyly. Losos [19] similarly indicated that the separation of Cuban twig anoles into two clades is not strongly supported by mtDNA phylogeny. The monophyly of twig Anoles was observed only when using nuclear markers (Additional file 2: Figure S2) and combining both mitochondrial and nuclear DNA (Fig. 2) but not when using only mitochondrial DNA (Additional file 2: Figure S2). This explains why Cuban twig Anoles were recovered as a polyphyletic group in the phylogeny of Cuban and non-Cuban species (Additional file 2: Figure S2) and highlights the importance of using multiple molecular markers to uncover the evolutionary relationship of the *Anolis* lizards. Accordingly, the present data suggest that each well-defined ecomorph class (crown-giant, trunk-crown, trunk-ground, and twig) are monophyletic, with the exception of grass-bush anoles. In contrasting results, a group of Cuban anoles that were previously regarded as unique [19] was divided into four different groups according to ecological and morphological similarities, and, with the exception of unique-type 2 anoles (three rock-dwelling species), these groups were all monophyletic, as shown previously [40, 46].

The present tree of divergence time estimates (Additional file 2: Figure S3) were based on a previous study of anole divergence [40], in which origins of ancestral species were estimated for all anoles at 95 Mya. However, taxon compositions and geographic sizes of sampling areas of the present Cuban *Anolis* species differ from those in the study by Nicholson [40], and require consideration in comparisons of trees. In contrast, age estimations of the main nodes in our tree were generally lower than those reported previously [40], and estimates of dating may be inaccurate [51]; however, the absolute species age was not necessary for the present analysis as their effects on genetic diversity can be used for examining the effect of species ages. The extinction of sister species is responsible for overestimating the age of existing species. Here, we covered most of the existing species of Cuban anoles; therefore, we consider that extinction of sister species has had little effect, although it is possible that some species’ ages might be overestimated owing to the extinction or missing sampling of sister species.

### Factors affecting interspecific differences in average genetic divergence among Cuban *Anolis* lizards

Our PGLS analyses suggest that species ages influence interspecific differences in genetic divergence among populations (Fig. 3), even after controlling for the effects of average geographic distances. In contrast, environmental heterogeneity within species ranges and ecomorph differences were not significantly associated with genetic divergence. Hence, older species have larger average genetic divergences, likely reflecting longer times to accumulate genetic divergence among populations. Accordingly, if gene flows between populations are very low, genetic divergence among populations will increase with time. In agreement, the present molecular phylogenic analysis indicates that, with few exceptions, individuals from the same or close locations form monophyletic groups, suggesting infrequent migration. Thus, when taxonomically older anole lizard species include older populations after divergence, species age becomes an important determinant of interspecific differences in genetic divergence among populations. Previous studies have related species age with geographical range sizes [11, 13, 14, 52], although several studies have reported unclear relationships between these variables [15, 53]. We identified a weak correlation between species age and range size (average geographic distances vs. species age, *r* = 0.0428, df = 24, *P* = 0.8356; PGLS, range = 61.43 age + 2354.09, *t* = 0.210, *P* = 0.8356), indicating that species range is not a covariate of species age.

A more accurate estimate of the genetic distance between populations requires intragenic variations using sufficient number of individuals within populations. To examine genetic divergence within species in our study, we examined 177 populations of 26 species; we usually used only one or few individuals for estimating genetic distances among populations. Both from a conservation viewpoint and because of technical difficulties, we could not collect sufficient numbers of samples within a population for all of the populations studied. Thus, our sampling strategy was to collect from several sampling locations to cover the largest possible proportion of the known geographic ranges for each species, and we collected randomly chosen one or few individuals from one population. Our aim was not to estimate the accurate values of genetic distance between populations, but to compare the relative degree of genetic divergence between populations among species. Our sampling procedure could reflect the average genetic divergence within species for species comparison. Further, in our studies, the examined genetic variations within populations would be not large. We used more than one sample (usually two) for 68 of 177 populations. The phylogeny of these individuals (Fig. 2) revealed that for 52 populations, the samples from the same populations were monophyletically related, so that genetic distances between samples within a population were smaller than those between the populations. Furthermore, seven of 16 populations wherein the samples within a population were paraphyletic; the samples from the same populations were genetically similar to those from geographically closer populations (~ < 50 km); therefore, our estimates of genetic distances were not largely affected by large intragenic population variation. Our study was based on the analysis of only few markers. Genome-wide data are essential for precise estimation of species age and genetic variations; further, we should also examine genetic divergence using genome-wide genetic markers in future studies.

We included geographic distance as a potential covariate of genetic distance in the present PGLS analyses, which indicated that geographic distance affects species genetic divergence. In addition, average geographic distances were highly correlated with species range sizes (*r* = 0.8509, *t* = 7.9375, *P* = 3.625 × 10^− 8^). Accordingly, PGLS analysis using species ranges instead of geographic distances showed that species ages and ranges significantly affect genetic divergence (Additional file 1: Table S5). Hence, although the effects of species range could not be separated from the effects of average measured geographic distances, species with larger species range may have larger genetic divergence. Fujisawa, Vogler and Barraclough [15] also showed that water beetle species with higher occupancy (i.e., a larger range size) were more genetically variable than species with lower occupancy, and suggested that species occupancy and range size are tightly correlated with population size, and that large population sizes are causal of genetic diversity [54, 55]. However, the relative contributions of population size and isolation-by-distance effects on genetic divergence were unclear in the present analyses.

Analyses of 19 climatic variables and percent tree cover found that species that inhabit more heterogeneous environments do not necessarily show higher levels of genetic divergence among populations based on 19 climatic variables and percent tree cover. In the present study, the number of individuals examined for some species was insufficient to conclude that environmental heterogeneity within species did not affect their genetic divergence; however, in some species, genetic divergence was lower, although environmental heterogeneity was high (e.g., Crown giant species such as *A. luteogularis* and *A. smallwoodi*). Thus, the results suggest that some *Anolis* species may not have adapted to local climatic differences, or local climate adaptations may not be related to divergence among populations when measured using neutral genetic markers. It has previously been hypothesized that *Anolis* lizard populations have adapted to environmental differences [19], wherein there should be variation in ecologically relevant traits among populations inhabiting different environments. For example, *Anolis* species that inhabit mountainous Lesser Antilles islands exhibit pronounced differences between populations, presumably following adaptations to different climates [56]. Moreover, in previous studies, geographic variations in ecologically relevant traits such as limb length, body shape, scalation, color, and body size have been associated with environmental variables such as temperature, humidity, and vegetation type [28, 57, 58]. However, Munoz et al. [59] demonstrated that color variations in *A. marmoratus* evolved in the absence of geographic isolation, and little or no genetic divergence was found among these populations using microsatellite loci. This could not eliminate the possibility that among Cuban *Anolis* species, local populations of a species can adapt to different climatic conditions without preventing substantial gene flow among the populations. Another study [6] showed that adaptive differences and selective pressures against dispersers play secondary roles in limiting gene flows between divergent habitats, constituting a small isolation-by-environment effect. However, these authors did not determine whether higher levels of environmental heterogeneity promote genetic divergence among habitats. The present results show that species that inhabit more heterogeneous environments do not necessarily have greater genetic divergence among populations.

In a study of inland snails, arboreal species had more divergent populations than ground-dwelling species [60], suggesting that species with different ecological niches have different patterns of genetic divergence among populations. Similarly, *Anolis* lizard species that belong to different ecomorphs may exhibit differing dispersal capacities due to divergence in morphology and microhabitat use, and these differences may be correlated with average genetic divergence. The present results could not find significant effects of ecomorph types on genetic divergence. However, we observed a tendency of crown-giant species to have lower genetic divergence than non-crown-giant species (0.0090 ± 0.0069 [SD] vs. 0.0467 ± 0.0207, respectively), suggesting that some ecomorphs’ characteristics affect genetic divergence. Accordingly, crown-giant species are clearly larger than other ecomorph species (mean male snout-vent length; 145.7 mm for crown-giant species and 34.8–59.18 for other ecomorph species), and may therefore cover greater distances and have greater dispersal ability. However, no significant effects of body size on genetic divergence were identified in PGLS analyses (*t* = − 1.1270, *P* = 0.2969). Further studies are required to consider whether other ecological or morphological characteristics affect genetic divergence among populations.

Phylogenies of trunk-ground and trunk-crown species usually show deep population divergence. For example, the trunk-ground species *A. allogus* is divided into two main clades, as shown herein and in a previous study [34]. Because one of these includes all eastern populations and the other comprises western and central populations, it has been suggested that these two clades should be considered as different species [34, 48]. Similarly, different paraphyletic clades have been identified in phylogenetic analyses of the two trunk-crown species *A. allisoni* and *A. porcatus*, again suggesting that eastern and western populations of *A. porcatus* should be distinguished at the species level based on genetic divergence [20]. Although we analyzed *A. allisoni* and *A. porcatus* as separate species, high genetic divergence was observed among populations of these clades, and deep population divergence was also observed among populations of *A. homolechis*, revealing unexpected levels of genetic variation within some *Anolis* species. Several previous studies have also showed deep interpopulation divergence among anoles from the Greater Antilles [20–26, 48] and Lesser Antilles [27–31], and in three Amazonian species [61]. Thus, our analyses indicate that the deep genetic divergences observed in several *Anolis* species could partly be driven by a longer isolation between populations.

## Conclusion

In this study, we performed phylogenetic analyses of Cuban *Anolis* lizards using unprecedented numbers of species, geographic samples, and molecular markers. The present analyses showed more parsimonious evolution of *Anolis* ecomorphs within Cuba and identified twig anoles as a monophyletic group. Subsequent PGLS analyses confirmed that species age is positively correlated with species’ average genetic divergence among populations. Although previous studies have focused on factors affecting genetic divergence within species, the present study for the first time showed that species differences in genetic divergence could be largely affected by species age. The generalizations of the present study warrant further examination in studies of Caribbean and mainland anoles. Moreover, studies of genetic divergence among species that belong to similar ecomorph classes are required to confirm the present assertions on other Greater Antilles islands.

## Additional files

Additional file 1: **Table S1. (a)** Location data and GenBank accession numbers of the Cuban specimens used in this study. **(b)** Regional sampling data. **Table S2**. GenBank sequence accession numbers of the non-Cuban specimens used in this study. **Table S3.** Description of Unique Type in Cuba Anoles. **Table S4.** Environmental variables for each locality. **Table S5.** Results of a phylogenetic generalized least squares (PGLS) analysis for the final model using both nuclear DNA (nDNA) and mitochondrial DNA (mtDNA) (a) and only mtDNA (b). (DOC 627 kb)

Additional file 2: **Figure S1.** Bayesian tree (50% majority consensus) based on three genes using 303 individuals from 33 Cuban *Anolis* species and two species from the genus *Leiocephalus*. **Figure S2.** Contrasting Bayesian phylogenies for nuclear DNA (based on ZNF521 and FBRSL1) and mtDNA (based on ND2) for Cuban anoles. **Figure S3.** Phylogenetic tree with estimates of divergence times based on the mitochondrial gene *ND2* (1036 bp). (DOC 8930 kb)
