# Supplementary material for: Factors affecting interspecific differences in genetic divergence among populations of Anolis lizards in Cuba
Source: Zoological Lett. 2018 Aug 9;4:21. doi: 10.1186/s40851-018-0107-x (PMC6085692; doi:10.1186/s40851-018-0107-x)
Supplement: Supplementary file 1 — Table S1. (a) Location data and GenBank accession numbers of the Cuban specimens used in this study. (b) Regional sampling data. Table S2. GenBank sequence accession numbers of the non-Cuban specimens used in this study. Table S3. Description of Unique Type in Cuba Anoles. Table S4. Environmental variables for each locality. Table S5. Results of a phylogenetic generalized least squares (PGLS) analysis for the final model using both nuclear DNA (nDNA) and mitochondrial DNA (mtDNA) (a) and only mtDNA (b). (DOC 627 kb) [file 40851_2018_107_MOESM1_ESM.doc]

**Additional file 1**

**Table S1** (a) Location data and GenBank accession numbers of the Cuban specimens used in this study; samples 1-232 were sequenced and their accession numbers begin with KT; samples 233-320 were sourced from Cádiz et al*.* (2013) and their accession numbers begin with AB; samples 321-323 were obtained from GenBank.

| **No.** | **Sample** | **Species** | **Location Code** | **Locality name** | **Ecomorph** | **NAD2** | **ZNF521** | **FBRSL1** |
| --- | --- | --- | --- | --- | --- | --- | --- | --- |
| 1 | 01_1_alu | *A. alutaceus* | C13 | Trinidad, Sancti Spiritus | Grass-Bush | 376179 | 375741 | 375960 |
| 2 | 01_2_alu | *A. alutaceus* | C12 | Manicaragua, Villa Clara | Grass-Bush | 376180 | 375742 | 375961 |
| 3 | 01_3_alu | *A. alutaceus* | C12 | Manicaragua, Villa Clara | Grass-Bush | 376181 | 375743 | 375962 |
| 4 | 01_4_alu | *A. alutaceus* | W10 | Candelaria, Artemisa, Cuba | Grass-Bush | 376182 | 375744 | 375963 |
| 5 | 01_5_alu | *A. alutaceus* | W10 | Candelaria, Artemisa, Cuba | Grass-Bush | 376183 | 375745 | 375964 |
| 6 | 01_6_alu | *A. alutaceus* | C14 | Sancti Spiritus, Sancti Spiritus | Grass-Bush | 376184 | 375746 | 375965 |
| 7 | 01_7_alu | *A. alutaceus* | W9 | Candelaria, Artemisa, Cuba | Grass-Bush | 376185 | 375747 | 375966 |
| 8 | 01_8_alu | *A. alutaceus* | W12 | Plaza de la Revolución, La Habana, Cuba | Grass-Bush | 376186 | 375748 | 375967 |
| 9 | 02_1_alu | *A. alutaceus* | W12 | Plaza de la Revolución, La Habana, Cuba | Grass-Bush | 376187 | 375749 | 375968 |
| 10 | 02_2_ine | *A. inexpectatus* | E32 | Baracoa, Guantanamo | Grass-Bush | 376188 | 375750 | 375969 |
| 11 | 02_3_ine | *A. inexpectatus* | E17 | Baracoa, Guantanamo | Grass-Bush | 376189 | 375751 | 375970 |
| 12 | 02_4_ine | *A. inexpectatus* | E17 | Baracoa, Guantanamo | Grass-Bush | 376190 | 375752 | 375971 |
| 13 | 02_5_ine | *A. inexpectatus* | E24 | Baracoa, Guantanamo | Grass-Bush | 376191 | 375753 | 375972 |
| 14 | 02_6_ine | *A. inexpectatus* | E24 | Baracoa, Guantanamo | Grass-Bush | 376192 | 375754 | 375973 |
| 15 | 02_7_ine | *A. inexpectatus* | E23 | Baracoa, Guantanamo | Grass-Bush | 376193 | 375755 | 375974 |
| 16 | 02_8_ine | *A. inexpectatus* | E23 | Baracoa, Guantanamo | Grass-Bush | 376194 | 375756 | 375975 |
| 17 | 03_2_ine | *A. inexpectatus* | E25 | Moa, Holguín | Grass-Bush | 376195 | 375757 | 375976 |
| 18 | 03_3_fug | *A. fugitivus* | E25 | Moa, Holguín | Grass-Bush | 376196 | 375758 | 375977 |
| 19 | 03_5_fug | *A. fugitivus* | E24 | Baracoa, Guantanamo | Grass-Bush | 376197 | 375759 | 375978 |
| 20 | 03_6_fug | *A. fugitivus* | E24 | Baracoa, Guantanamo | Grass-Bush | 376198 | 375760 | 375979 |
| 21 | 03_7_cup | *A. cupeyalensis* | E27 | Yateras, Guantánamo | Grass-Bush | 376199 | 375761 | 375980 |
| 22 | 03_8_cup | *A. cupeyalensis* | E27 | Yateras, Guantánamo | Grass-Bush | 376200 | 375762 | 375981 |
| 23 | 04_3_rej | *A. rejectus* | E5 | Santiago de Cuba, Santiago de Cuba | Grass-Bush | 376201 | - | - |
| 24 | 04_5_van | *A. vanidicus* | C13 | Trinidad, Sancti Spiritus | Grass-Bush | 376202 | - | - |
| 25 | 04_7_anf | *A. anfiloquioi* | E19 | Baracoa, Guantanamo | Grass-Bush | 376203 | - | - |
| 26 | 04_8_cya | *A. cyanopleurus* | E25 | Moa, Holguín | Grass-Bush | 376204 | 375763 | 375982 |
| 27 | 05_1_cya | *A. cyanopleurus* | E25 | Moa, Holguín | Grass-Bush | 376205 | 375764 | 375983 |
| 28 | 05_2_cya | *A. cyanopleurus* | E19 | Baracoa, Guantanamo | Grass-Bush | 376206 | 375765 | 375984 |
| 29 | 05_3_ang | *A. angusticeps* | W1 | Sandino, Pinar del Río, Cuba | Twig | 376207 | 375766 | 375985 |
| 30 | 05_7_ang | *A. angusticeps* | C16 | Trinidad, Sancti Spiritus | Twig | 376208 | 375767 | 375986 |
| 31 | 06_1_ang | *A. angusticeps* | W12 | Plaza de la Revolución, La Habana, Cuba | Twig | 376209 | 375768 | 375987 |
| 32 | 06_2_ang | *A. angusticeps* | W12 | Plaza de la Revolución, La Habana, Cuba | Twig | 376210 | 375769 | 375988 |
| 33 | 06_3_ang | *A. angusticeps* | W10 | Candelaria, Artemisa, Cuba | Twig | 376211 | 375770 | 375989 |
| 34 | 06_4_ang | *A. angusticeps* | W10 | Candelaria, Artemisa, Cuba | Twig | 376212 | 375771 | 375990 |
| 35 | 06_7_ang | *A. angusticeps* | C8 | Cienaga de Zapata, Matanzas | Twig | 376213 | 375772 | 375991 |
| 36 | 06_8_ang | *A. angusticeps* | C8 | Cienaga de Zapata, Matanzas | Twig | 376214 | 375773 | 375992 |
| 37 | 07_1_ang | *A. angusticeps* | C2 | Santa Cruz del Norte, Mayabeque | Twig | 376215 | 375774 | 375993 |
| 38 | 07_2_ang | *A. angusticeps* | C2 | Santa Cruz del Norte, Mayabeque | Twig | 376216 | 375775 | 375994 |
| 39 | 07_3_ang | *A. angusticeps* | E1 | Niquero, Granma | Twig | 376217 | 375776 | 375995 |
| 40 | 07_4_ang | *A. angusticeps* | E1 | Niquero, Granma | Twig | 376218 | 375777 | 375996 |
| 41 | 07_5_ang | *A. angusticeps* | C1 | Cárdenas, Matanzas | Twig | 376219 | 375778 | 375997 |
| 42 | 07_6_ang | *A. angusticeps* | C1 | Cárdenas, Matanzas | Twig | 376220 | 375779 | 375998 |
| 43 | 07_7_pum | *A. pumilus* | W1 | Sandino, Pinar del Río, Cuba | Unique Type 1 | 376221 | 375780 | 375999 |
| 44 | 07_8_pum | *A. pumilus* | C8 | Cienaga de Zapata, Matanzas | Unique Type 1 | 376222 | 375781 | 376000 |
| 45 | 08_1_pum | *A. pumilus* | C18 | Sierra de Cubitas, Camaguey | Unique Type 1 | 376223 | 375782 | 376001 |
| 46 | 08_2_pum | *A. pumilus* | C18 | Sierra de Cubitas, Camaguey | Unique Type 1 | 376224 | 375783 | 376002 |
| 47 | 08_3_ala | *A. alayoni* | E19 | Baracoa, Guantanamo | Twig | 376225 | 375784 | 376003 |
| 48 | 08_4_ala | *A. alayoni* | E17 | Baracoa, Guantanamo | Twig | 376226 | 375785 | 376004 |
| 49 | 08_5_ala | *A. alayoni* | E3 | Niquero, Granma | Twig | 376227 | 375786 | 376005 |
| 50 | 08_7_argi | *A. argillaceus* | E13 | San Antonio del Sur, Guantánamo | Unique Type 1 | 376228 | 375787 | 376006 |
| 51 | 08_8_argi | *A. argillaceus* | E18 | Baracoa, Guantanamo | Unique Type 1 | 376229 | 375788 | 376007 |
| 52 | 09_1_argi | *A. argillaceus* | E27 | Yateras, Guantánamo | Unique Type 1 | 376230 | 375789 | 376008 |
| 53 | 09_6_rui | *A. ruibali* | E1 | Niquero, Granma | Unique Type 1 | 376231 | - | - |
| 54 | 09_7_guaz | *A. guazuma* | E5 | Santiago de Cuba, Santiago de Cuba | Twig | 376232 | 375790 | 376009 |
| 55 | 10_3_loy | *A. loysiana* | W10 | Candelaria, Artemisa, Cuba | Unique Type 1 | 376233 | 375791 | 376010 |
| 56 | 10_4_loy | *A. loysiana* | W10 | Candelaria, Artemisa, Cuba | Unique Type 1 | 376234 | 375792 | 376011 |
| 57 | 10_5_arg | *A. argenteolus* | E3 | Niquero, Granma | Unique Type 2 | 376235 | 375793 | 376012 |
| 58 | 10_6_arg | *A. argenteolus* | E3 | Niquero, Granma | Unique Type 2 | 376236 | 375794 | 376013 |
| 59 | 10_7_arg | *A. argenteolus* | E20 | Baracoa, Guantanamo | Unique Type 2 | 376237 | 375795 | 376014 |
| 60 | 10_8_arg | *A. argenteolus* | E20 | Baracoa, Guantanamo | Unique Type 2 | 376238 | 375796 | 376015 |
| 61 | 11_1_arg | *A. argenteolus* | E18 | Baracoa, Guantanamo | Unique Type 2 | 376239 | 375797 | 376016 |
| 62 | 11_2_arg | *A. argenteolus* | E18 | Baracoa, Guantanamo | Unique Type 2 | 376240 | 375798 | 376017 |
| 63 | 11_3_arg | *A. argenteolus* | E36 | San Antonio del Sur, Guantánamo | Unique Type 2 | 376241 | 375799 | 376018 |
| 64 | 11_4_arg | *A. argenteolus* | E36 | San Antonio del Sur, Guantánamo | Unique Type 2 | 376242 | 375800 | 376019 |
| 65 | 11_5_arg | *A. argenteolus* | E11 | Guamá, Santiago de Cuba | Unique Type 2 | 376243 | 375801 | 376020 |
| 66 | 11_6_arg | *A. argenteolus* | E11 | Guamá, Santiago de Cuba | Unique Type 2 | 376244 | 375802 | 376021 |
| 67 | 11_7_arg | *A. argenteolus* | E17 | Baracoa, Guantanamo | Unique Type 2 | 376245 | 375803 | 376022 |
| 68 | 11_8_arg | *A. argenteolus* | E17 | Baracoa, Guantanamo | Unique Type 2 | 376246 | 375804 | 376023 |
| 69 | 12_1_arg | *A. argenteolus* | E1 | Niquero, Granma | Unique Type 2 | 376247 | 375805 | 376024 |
| 70 | 12_2_arg | *A. argenteolus* | E1 | Niquero, Granma | Unique Type 2 | 376248 | 375806 | 376025 |
| 71 | 12_3_luc | *A. lucius* | C2 | Santa Cruz del Norte, Mayabeque | Unique Type 2 | 376249 | 375807 | 376026 |
| 72 | 12_4_luc | *A. lucius* | W13 | Güines, Mayabeque, Cuba | Unique Type 2 | 376250 | 375808 | 376027 |
| 73 | 12_5_luc | *A. lucius* | W13 | Güines, Mayabeque, Cuba | Unique Type 2 | 376251 | 375809 | 376028 |
| 74 | 12_6_luc | *A. lucius* | C18 | Sierra de Cubitas, Camaguey | Unique Type 2 | 376252 | 375810 | 376029 |
| 75 | 12_7_luc | *A. lucius* | C18 | Sierra de Cubitas, Camaguey | Unique Type 2 | 376253 | 375811 | 376030 |
| 76 | 12_8_luc | *A. lucius* | C16 | Trinidad, Sancti Spiritus | Unique Type 2 | 376254 | 375812 | 376031 |
| 77 | 13_1_luc | *A. lucius* | C16 | Trinidad, Sancti Spiritus | Unique Type 2 | 376255 | 375813 | 376032 |
| 78 | 13_2_luc | *A. lucius* | C15 | Cumanayagua, Cienfuegos | Unique Type 2 | 376256 | 375814 | 376033 |
| 79 | 13_3_luc | *A. lucius* | C15 | Cumanayagua, Cienfuegos | Unique Type 2 | 376257 | 375815 | 376034 |
| 80 | 13_4_luc | *A. lucius* | C12 | Manicaragua, Villa Clara | Unique Type 2 | 376258 | 375816 | 376035 |
| 81 | 13_6_alli | *A. allisoni* | E3 | Niquero, Granma | Trunk-Crown | 376259 | 375817 | 376036 |
| 82 | 13_7_alli | *A. allisoni* | E3 | Niquero, Granma | Trunk-Crown | 376260 | 375818 | 376037 |
| 83 | 13_8_alli | *A. allisoni* | E1 | Niquero, Granma | Trunk-Crown | 376261 | 375819 | 376038 |
| 84 | 14_1_por | *A. porcatus* | E20 | Baracoa, Guantanamo | Trunk-Crown | 376262 | 375820 | 376039 |
| 85 | 14_2_alli | *A. allisoni* | E1 | Niquero, Granma | Trunk-Crown | 376263 | 375821 | 376040 |
| 86 | 14_3_por | *A. porcatus* | E26 | Yateras, Guantánamo | Trunk-Crown | 376264 | 375822 | 376041 |
| 87 | 14_4_por | *porcatus* | C13 | Trinidad, Sancti Spiritus | Trunk-Crown | 376265 | 375823 | 376042 |
| 88 | 14_5_por | *A. porcatus* | C13 | Trinidad, Sancti Spiritus | Trunk-Crown | 376266 | 375824 | 376043 |
| 89 | 14_6_por | *A. porcatus* | E18 | Baracoa, Guantanamo | Trunk-Crown | 376267 | 375825 | 376044 |
| 90 | 14_7_por | *A. porcatus* | E25 | Moa, Holguín | Trunk-Crown | 376268 | 375826 | 376045 |
| 91 | 14_8_por | *A. porcatus* | E25 | Moa, Holguín | Trunk-Crown | 376269 | 375827 | 376046 |
| 92 | 15_1_alli | *A. allisoni* | C6 | Jagüey Grande, Matanzas | Trunk-Crown | 376270 | 375828 | 376047 |
| 93 | 15_2_alli | *A. allisoni* | C5 | Ciénaga de Zapata, Matanzas | Trunk-Crown | 376271 | 375829 | 376048 |
| 94 | 15_3_alli | *A. allisoni* | C5 | Ciénaga de Zapata, Matanzas | Trunk-Crown | 376272 | 375830 | 376049 |
| 95 | 15_5_alli | *A. allisoni* | C7 | Cienaga de Zapata, Matanzas | Trunk-Crown | 376273 | 375831 | 376050 |
| 96 | 15_6_alli | *A. allisoni* | C18 | Sierra de Cubitas, Camaguey | Trunk-Crown | 376274 | 375832 | 376051 |
| 97 | 15_7_alli | *A. allisoni* | C18 | Sierra de Cubitas, Camaguey | Trunk-Crown | 376275 | 375833 | 376052 |
| 98 | 15_8_equ | *A. equestris* | C1 | Cárdenas, Matanzas | Crown-Giant | 376276 | 375834 | 376053 |
| 99 | 16_2_lut | *A. luteogularis* | W12 | Plaza de la Revolución, La Habana, Cuba | Crown-Giant | 376277 | 375835 | 376054 |
| 100 | 16_3_lut | *A. luteogularis* | W12 | Plaza de la Revolución, La Habana, Cuba | Crown-Giant | 376278 | 375836 | 376055 |
| 101 | 16_7_equ | *A. equestris* | C13 | Trinidad, Sancti Spiritus | Crown-Giant | 376279 | 375837 | 376056 |
| 102 | 16_8_sma | *A. smallwoodi* | E25 | Moa, Holguín | Crown-Giant | 376280 | 375838 | 376057 |
| 103 | 17_3_barb | *A. barbatus* | W10 | Candelaria, Artemisa, Cuba | Unique Type 4 | 376281 | 375839 | 376058 |
| 104 | 17_8_ang | *A. angusticeps* | W9 | Candelaria, Artemisa, Cuba | Twig | 376282 | 375840 | 376059 |
| 105 | 18_2_alu | *A. alutaceus* | W9 | Candelaria, Artemisa, Cuba | Grass-Bush | 376283 | 375841 | 376060 |
| 106 | 18_3_lut | *A. luteogularis* | W9 | Candelaria, Artemisa, Cuba | Crown-Giant | 376284 | 375842 | 376061 |
| 107 | 18_4_por | *A. porcatus* | W9 | Candelaria, Artemisa, Cuba | Trunk-Crown | 376285 | 375843 | 376062 |
| 108 | 18_5_por | *A. porcatus* | W9 | Candelaria, Artemisa, Cuba | Trunk-Crown | 376286 | 375844 | 376063 |
| 109 | 18_6_bart | *A. bartchi* | W14 | Viñales, Pinar del Río, Cuba | Unique Type 2 | 376287 | 375845 | 376064 |
| 110 | 18_8_bart | *A. bartchi* | W5 | Viñales, Pinar del Río, Cuba | Unique Type 2 | 376288 | 375846 | 376065 |
| 111 | 19_1_alu | *A. alutaceus* | W5 | Viñales, Pinar del Río, Cuba | Grass-Bush | 376289 | 375847 | 376066 |
| 112 | 19_2_alu | *A. alutaceus* | W5 | Viñales, Pinar del Río, Cuba | Grass-Bush | 376290 | 375848 | 376067 |
| 113 | 19_3_por | *A. porcatus* | W5 | Viñales, Pinar del Río, Cuba | Trunk-Crown | 376291 | 375849 | 376068 |
| 114 | 19_4_por | *A. porcatus* | W5 | Viñales, Pinar del Río, Cuba | Trunk-Crown | 376292 | 375850 | 376069 |
| 115 | 19_5_barb | *A. barbatus* | W9 | Candelaria, Artemisa, Cuba | Unique Type 4 | 376293 | 375851 | 376070 |
| 116 | 19_6_barb | *A. barbatus* | W9 | Candelaria, Artemisa, Cuba | Unique Type 4 | 376294 | 375852 | 376071 |
| 117 | 19_7_por | *A. porcatus* | W12 | Plaza de la Revolución, La Habana, Cuba | Trunk-Crown | 376295 | 375853 | 376072 |
| 118 | 19_8_por | *A. porcatus* | W12 | Plaza de la Revolución, La Habana, Cuba | Trunk-Crown | 376296 | 375854 | 376073 |
| 119 | 20_2_bar | *A. barbatus* | E19 | Baracoa, Guantanamo | Unique Type 4 | 376297 | - | - |
| 120 | 20_3_equ | *A. equestris* | C6 | Jagüey Grande, Matanzas | Crown-Giant | 376298 | 375855 | 376074 |
| 121 | 20_4_equ | *A. equestris* | C6 | Jagüey Grande, Matanzas | Crown-Giant | 376299 | 375856 | 376075 |
| 122 | 20_5_ver | *A. vermiculatus* | W10 | Candelaria, Artemisa, Cuba | Unique Type 3 | 376300 | - | - |
| 123 | 21_2_alu | *A. alutaceus* | W1 | Sandino, Pinar del Río, Cuba | Grass-Bush | 376301 | 375857 | 376076 |
| 124 | 21_3_alu | *A. alutaceus* | W1 | Sandino, Pinar del Río, Cuba | Grass-Bush | 376302 | 375858 | 376077 |
| 125 | 21_4_alu | *A. alutaceus* | E5 | Santiago de Cuba, Santiago de Cuba | Grass-Bush | 376303 | 375859 | 376078 |
| 126 | 21_5_alu | *A. alutaceus* | E5 | Santiago de Cuba, Santiago de Cuba | Grass-Bush | 376304 | 375860 | 376079 |
| 127 | 21_7_alu | *A. alutaceus* | C18 | Sierra de Cubitas, Camaguey | Grass-Bush | 376305 | 375861 | 376080 |
| 128 | 21_8_alu | *A. alutaceus* | C3 | Madruga, Mayabeque | Grass-Bush | 376306 | 375862 | 376081 |
| 129 | 22_1_alu | *A. alutaceus* | C3 | Madruga, Mayabeque | Grass-Bush | 376307 | 375863 | 376082 |
| 130 | 22_4_alu | *A. alutaceus* | C13 | Trinidad, Sancti Spiritus | Grass-Bush | 376308 | 375864 | 376083 |
| 131 | 22_5_argi | *A. argillaceus* | E20 | Baracoa, Guantanamo | Unique Type 1 | 376309 | 375865 | 376084 |
| 132 | 22_6_argi | *A. argillaceus* | E27 | Yateras, Guantánamo | Unique Type 1 | 376310 | 375866 | 376085 |
| 133 | 22_7_argi | *A. argillaceus* | E20 | Baracoa, Guantanamo | Unique Type 1 | 376311 | 375867 | 376086 |
| 134 | 23_3_alf | *A. alfaroi* | E31 | Yateras, Guantánamo | Grass-Bush | 376312 | - | - |
| 135 | 23_4_iso | *A. isolepis* | E31 | Yateras, Guantánamo | Trunk-Crown | 376313 | 375868 | 376087 |
| 136 | 23_5_iso | *A. isolepis* | E31 | Yateras, Guantánamo | Trunk-Crown | 376314 | 375869 | 376088 |
| 137 | 23_8_cya | *A. cyanopleurus* | E14 | Imias, Guantanamo | Grass-Bush | 376315 | 375870 | 376089 |
| 138 | 24_3_spe | *A. spectrum* | C21 | Carlos Rojas, Matanzas | Grass-Bush | 376316 | - | - |
| 139 | 24_4_cup | *A. cupeyalensis* | E31 | Yateras, Guantánamo | Grass-Bush | 376317 | 375871 | 376090 |
| 140 | 24_5_cya | *A. cyanopleurus* | E14 | Imias, Guantanamo | Grass-Bush | 376318 | 375872 | 376091 |
| 141 | 24_6_cya | *A. cyanopleurus* | E14 | Imias, Guantanamo | Grass-Bush | 376319 | 375873 | 376092 |
| 142 | 25_1_argi | *A. argillaceus* | E12 | San Antonio del Sur, Guantánamo | Unique Type 1 | 376320 | 375874 | 376093 |
| 143 | 25_2_argi | *A. argillaceus* | E12 | San Antonio del Sur, Guantánamo | Unique Type 1 | 376321 | 375875 | 376094 |
| 144 | 25_3_argi | *A. argillaceus* | E12 | San Antonio del Sur, Guantánamo | Unique Type 1 | 376322 | 375876 | 376095 |
| 145 | 25_4_lit | *A. litoralis* | E8 | Niceto Pérez, Guantánamo | Unique Type 1 | 376323 | - | - |
| 146 | 25_6_pum | *A. pumilus* | C9 | Martí, Matanzas | Unique Type 1 | 376324 | 375877 | 376096 |
| 147 | 25_7_pum | *A. pumilus* | C9 | Martí, Matanzas | Unique Type 1 | 376325 | 375878 | 376097 |
| 148 | 25_8_cya | *A. cyanopleurus* | E15 | Maisí, Guantánamo | Grass-Bush | 376326 | 375879 | 376098 |
| 149 | 26_1_sma | *A. smallwoodi* | E31 | Yateras, Guantánamo | Crown-Giant | 376327 | 375880 | 376099 |
| 150 | 26_2_sma | *A. smallwoodi* | E31 | Yateras, Guantánamo | Crown-Giant | 376328 | 375881 | 376100 |
| 151 | 26_3_sma | *A. smallwoodi* | E8 | Niceto Pérez, Guantánamo | Crown-Giant | 376329 | 375882 | 376101 |
| 152 | 26_4_sma | *A. smallwoodi* | E12 | San Antonio del Sur, Guantánamo | Crown-Giant | 376330 | 375883 | 376102 |
| 153 | 26_5_equ | *A. equestris* | C4 | Santa Cruz del Norte, Mayabeque | Crown-Giant | 376331 | 375884 | 376103 |
| 154 | 26_6_equ | *A. equestris* | C4 | Santa Cruz del Norte, Mayabeque | Crown-Giant | 376332 | 375885 | 376104 |
| 155 | 26_7_equ | *A. equestris* | C11 | Ciénaga de Zapata, Matanzas | Crown-Giant | 376333 | 375886 | 376105 |
| 156 | 27_2_equ | *A. equestris* | C10 | Ciénaga de Zapata, Matanzas | Crown-Giant | 376334 | 375887 | 376106 |
| 157 | 27_5_lut | *A. luteogularis* | W10 | Candelaria, Artemisa, Cuba | Crown-Giant | 376335 | 375888 | 376107 |
| 158 | 27_6_equ | *A. equestris* | C18 | Sierra de Cubitas, Camaguey | Crown-Giant | 376336 | 375889 | 376108 |
| 159 | 27_7_equ | *A. equestris* | C20 | Yaguajay, Sancti Spiritus | Crown-Giant | 376337 | 375890 | 376109 |
| 160 | 27_8_lut | *A. luteogularis* | W12 | Plaza de la Revolución, La Habana, Cuba | Crown-Giant | 376338 | 375891 | 376110 |
| 161 | 28_1_lut | *A. luteogularis* | W10 | Candelaria, Artemisa, Cuba | Crown-Giant | 376339 | 375892 | 376111 |
| 162 | 28_6_ine | *A. inexpectatus* | E31 | Yateras, Guantánamo | Grass-Bush | 376340 | 375893 | 376112 |
| 163 | 30_1_cha | *A. chamaleonides* | E6 | Bartolomé Masó, Granma | Unique Type 4 | 376341 | 375894 | 376113 |
| 164 | 30_2_nob | *A. noblei* | E6 | Bartolomé Masó, Granma | Crown-Giant | 376342 | 375895 | 376114 |
| 165 | 30_3_nob | *A. noblei* | E7 | Tercer Frente, Santiago de Cuba | Crown-Giant | 376343 | 375896 | 376115 |
| 166 | 30_6_sma | *A. smallwoodi* | E33 | Moa, Holguín | Crown-Giant | 376344 | 375897 | 376116 |
| 167 | 30_7_ang | *A. angusticeps* | E35 | Gibara, Holguín | Twig | 376345 | 375898 | 376117 |
| 168 | 30_8_iso | *A. isolepis* | E35 | Gibara, Holguín | Trunk-Crown | 376346 | 375899 | 376118 |
| 169 | 31_1_iso | *A. isolepis* | E35 | Gibara, Holguín | Trunk-Crown | 376347 | 375900 | 376119 |
| 170 | 31_3_alt | *A. altitudinalis* | E10 | Guamá, Santiago de Cuba | Trunk-Crown | 376348 | - | - |
| 171 | 31_4_cen | *A. centralis* | E35 | Gibara, Holguín | Unique Type 1 | 376349 | - | - |
| 172 | 31_8_guaz | *A. guazuma* | E6 | Bartolomé Masó, Granma | Twig | 376350 | 375901 | 376120 |
| 173 | 32_1_luc | *A. lucius* | E35 | Gibara, Holguín | Unique Type 2 | 376351 | 375902 | 376121 |
| 174 | 32_2_alu | *A. alutaceus* | E7 | Tercer Frente, Santiago de Cuba | Grass-Bush | 376352 | 375903 | 376122 |
| 175 | 32_3_guaz | *A. guazuma* | E7 | Tercer Frente, Santiago de Cuba | Twig | 376353 | 375904 | 376123 |
| 176 | 32_4_iso | *A. isolepis* | E7 | Tercer Frente, Santiago de Cuba | Trunk-Crown | 376354 | 375905 | 376124 |
| 177 | 32_5_cha | *A. chamaleonides* | E10 | Guamá, Santiago de Cuba | Unique Type 4 | 376355 | 375906 | 376125 |
| 178 | 33_1_loy | *A. loysiana* | E10 | Guamá, Santiago de Cuba | Unique Type 1 | 376356 | 375907 | 376126 |
| 179 | 33_5_lut | *A. luteogularis* | W8 | Sandino, Pinar del Río, Cuba | Crown-Giant | 376357 | 375908 | 376127 |
| 180 | 33_6_lut | *A. luteogularis* | W1 | Sandino, Pinar del Río, Cuba | Crown-Giant | 376358 | 375909 | 376128 |
| 181 | 34_1_por | *A. porcatus* | W1 | Sandino, Pinar del Río, Cuba | Trunk-Crown | 376359 | 375910 | 376129 |
| 182 | 34_3_qua | *A. quadriocelifer* | W2 | Sandino, Pinar del Río, Cuba | Trunk-Ground | 376360 | 375911 | 376130 |
| 183 | 34_4_cha | *A. chamaleonides* | C9 | Martí, Matanzas | Unique Type 4 | 376361 | 375912 | 376131 |
| 184 | 35_3_sag | *A. sagrei* | C17 | Trinidad, Sancti Spiritus | Trunk-Ground | 376363 | 375914 | 376133 |
| 185 | 35_5_guam | *A. guamuhaya* | C13 | Trinidad, Sancti Spiritus | Unique Type 4 | 376364 | - | - |
| 186 | 35_7_loy | *A. loysiana* | W1 | Sandino, Pinar del Río, Cuba | Unique Type 1 | 376365 | 375915 | 376134 |
| 187 | 36_5_pum | *A. pumilus* | C2 | Santa Cruz del Norte, Mayabeque | Unique Type 1 | 376367 | 375917 | 376136 |
| 188 | 36_6_pum | *A. pumilus* | C2 | Santa Cruz del Norte, Mayabeque | Unique Type 1 | 376368 | 375918 | 376137 |
| 189 | 38_3_nob | *A. noblei* | E6 | Bartolomé Masó, Granma | Crown-Giant | 376369 | 375919 | 376138 |
| 190 | 38_4_alu | *A. alutaceus* | E6 | Bartolomé Masó, Granma | Grass-Bush | 376370 | 375920 | 376139 |
| 191 | 38_5_alu | *A. alutaceus* | E6 | Bartolomé Masó, Granma | Grass-Bush | 376371 | 375921 | 376140 |
| 192 | 39_8_cli | *A. clivicola* | E10 | Guamá, Santiago de Cuba | Grass-Bush | 376372 | - | - |
| 193 | 40_3_argen | *A. argenteolus* | E7 | Tercer Frente, Santiago de Cuba | Unique Type 2 | 376373 | 375922 | 376141 |
| 194 | 40_4_argen | *A. argenteolus* | E7 | Tercer Frente, Santiago de Cuba | Unique Type 2 | 376374 | 375923 | 376142 |
| 195 | 40_5_all | *A. allogus* | E7 | Tercer Frente, Santiago de Cuba | Trunk-Ground | 376375 | 375924 | 376143 |
| 196 | 40_6_all | *A. allogus* | E7 | Tercer Frente, Santiago de Cuba | Trunk-Ground | 376376 | 375925 | 376144 |
| 197 | 41_1_all | *A. allogus* | E10 | Guamá, Santiago de Cuba | Trunk-Ground | 376377 | 375926 | 376145 |
| 198 | 41_2_hom | *A. homolechis* | E7 | Tercer Frente, Santiago de Cuba | Trunk-Ground | 376378 | 375927 | 376146 |
| 199 | 41_4_hom | *A. homolechis* | E10 | Guamá, Santiago de Cuba | Trunk-Ground | 376379 | 375928 | 376147 |
| 200 | 41_5_hom | *A. homolechis* | E10 | Guamá, Santiago de Cuba | Trunk-Ground | 376380 | 375929 | 376148 |
| 201 | 41_7_bre | *A. bremeri* | W8 | Sandino, Pinar del Río, Cuba | Trunk-Ground | 376381 | 375930 | 376149 |
| 202 | 41_8_sag | *A. sagrei* | W8 | Sandino, Pinar del Río, Cuba | Trunk-Ground | 376382 | 375931 | 376150 |
| 203 | 42_1_bre | *A. bremeri* | W8 | Sandino, Pinar del Río, Cuba | Trunk-Ground | 376383 | 375932 | 376151 |
| 204 | 42_6_pum | *A. pumilus* | W8 | Sandino, Pinar del Río, Cuba | Unique Type 1 | 376384 | 375933 | 376152 |
| 205 | 42_7_pum | *A. pumilus* | W8 | Sandino, Pinar del Río, Cuba | Unique Type 1 | 376385 | 375934 | 376153 |
| 206 | 42_8_lut | *A. luteogularis* | W8 | Sandino, Pinar del Río, Cuba | Crown-Giant | 376386 | 375935 | 376154 |
| 207 | 43_2_lut | *A. luteogularis* | W1 | Sandino, Pinar del Río, Cuba | Crown-Giant | 376387 | 375936 | 376155 |
| 208 | 43_5_hom | *A. homolechis* | W2 | Sandino, Pinar del Río, Cuba | Trunk-Ground | 376388 | 375937 | 376156 |
| 209 | 43_6_qua | *A. quadriocelifer* | W2 | Sandino, Pinar del Río, Cuba | Trunk-Ground | 376389 | 375938 | 376157 |
| 210 | 44_1_sag | *A. sagrei* | C17 | Trinidad, Sancti Spiritus | Trunk-Ground | 376390 | 375939 | 376158 |
| 211 | 44_2_sag | *A. sagrei* | C17 | Trinidad, Sancti Spiritus | Trunk-Ground | 376391 | 375940 | 376159 |
| 212 | 44_5_jub | *A. jubar* | E16 | San Antonio del Sur, Guantánamo | Trunk-Ground | 376392 | 375941 | 376160 |
| 213 | 44_6_jub | *A. jubar* | E16 | San Antonio del Sur, Guantánamo | Trunk-Ground | 376393 | 375942 | 376161 |
| 214 | 45_4_sag | *A. sagrei* | C3 | Madruga, Mayabeque | Trunk-Ground | 376394 | 375943 | 376162 |
| 215 | 45_5_sag | *A. sagrei* | C3 | Madruga, Mayabeque | Trunk-Ground | 376395 | 375944 | 376163 |
| 216 | 45_6_hom | *A. homolechis* | C3 | Madruga, Mayabeque | Trunk-Ground | 376396 | 375945 | 376164 |
| 217 | 45_7_hom | *A. homolechis* | C3 | Madruga, Mayabeque | Trunk-Ground | 376397 | 375946 | 376165 |
| 218 | 46_1_all | *A. allogus* | C3 | Madruga, Mayabeque | Trunk-Ground | 376398 | 375947 | 376166 |
| 219 | 46_2_all | *A. allogus* | C3 | Madruga, Mayabeque | Trunk-Ground | 376399 | 375948 | 376167 |
| 220 | 46_7_qua | *A. quadriocelifer* | W1 | Sandino, Pinar del Río, Cuba | Trunk-Ground | 376400 | 375949 | 376168 |
| 221 | 47_3_small | *A. smallwoodi* | E25 | Moa, Holguín | Crown-Giant | 376401 | 375950 | 376169 |
| 222 | 48_1_hom | *A. homolechis* | E34 | Manuel Tames, Guantánamo | Trunk-Ground | 376402 | 375951 | 376170 |
| 223 | 48_2_hom | *A. homolechis* | E34 | Manuel Tames, Guantánamo | Trunk-Ground | 376403 | 375952 | 376171 |
| 224 | 49_2_hom | *A. homolechis* | E8 | Niceto Pérez, Guantánamo | Trunk-Ground | 376404 | 375953 | 376172 |
| 225 | 49_3_hom | *A. homolechis* | E8 | Niceto Pérez, Guantánamo | Trunk-Ground | 376405 | 375954 | 376173 |
| 226 | 54_1_jub | *A. jubar* | E15 | Maisí, Guantánamo | Trunk-Ground | 376406 | 375955 | 376174 |
| 227 | 54_2_jub | *A. jubar* | E15 | Maisí, Guantánamo | Trunk-Ground | 376407 | 375956 | 376175 |
| 228 | 54_7_all | *A. allogus* | E15 | Maisí, Guantánamo | Trunk-Ground | 376408 | 375957 | 376176 |
| 229 | 54_8_all | *A. allogus* | E15 | Maisí, Guantánamo | Trunk-Ground | 376409 | 375958 | 376177 |
| 230 | 55_3_bre | *A. bremeri* | W6 | Viñales, Pinar del Río, Cuba | Trunk-Ground | 376410 | 375959 | 376178 |
| 231 | 35_1_L. raviceps | *Leiocephalus raviceps* | C1 | Cárdenas, Matanzas | - | 376362 | 375913 | 376132 |
| 232 | 36_1_L. onaneyi | *Leiocephalus onaneyi* | E13 | San Antonio del Sur, Guantánamo | - | 376366 | 375916 | 376135 |
| 233 | 01_1_all | *A. allogus* | E18 | Baracoa, Guantanamo | Trunk-Ground | 712812 | 710477 | 710783 |
| 234 | 01_4_all | *A. allogus* | W3 | Minas de Matahambre, Pinar del Río, Cuba | Trunk-Ground | 712815 | 710478 | 710784 |
| 235 | 01_6_bre | *A. bremeri* | W3 | Minas de Matahambre, Pinar del Río, Cuba | Trunk-Ground | 712817 | 710479 | 710785 |
| 236 | 02_4_hom | *A. homolechis* | C2 | Santa Cruz del Norte, Mayabeque | Trunk-Ground | 712823 | 710480 | 710786 |
| 237 | 02_7_hom | *A. homolechis* | W4 | Minas de Matahambre, Pinar del Río, Cuba | Trunk-Ground | 712826 | 710481 | 710787 |
| 238 | 03_2_hom | *A. homolechis* | C12 | Manicaragua, Villa Clara | Trunk-Ground | 712829 | 710482 | 710788 |
| 239 | 04_2_hom | *A. homolechis* | E23 | Baracoa, Guantanamo | Trunk-Ground | 712837 | 710484 | 710790 |
| 240 | 04_3_hom | *A. homolechis* | W3 | Minas de Matahambre, Pinar del Río, Cuba | Trunk-Ground | 712838 | 710485 | 710791 |
| 241 | 04_5_hom | *A. homolechis* | C13 | Trinidad, Sancti Spiritus | Trunk-Ground | 712840 | 710486 | 710792 |
| 242 | 05_2_hom | *A. homolechis* | C1 | Cárdenas, Matanzas | Trunk-Ground | 712845 | 710487 | 710793 |
| 243 | 05_3_mes | *A. mestrei* | W3 | Minas de Matahambre, Pinar del Río, Cuba | Trunk-Ground | 712846 | 710488 | 710794 |
| 244 | 06_1_rub | *A. rubribarbus* | E24 | Baracoa, Guantanamo | Trunk-Ground | 712852 | 710489 | 710795 |
| 245 | 06_2_sag | *A. sagrei* | E18 | Baracoa, Guantanamo | Trunk-Ground | 712853 | 710490 | 710796 |
| 246 | 06_3_sag | *A. sagrei* | C15 | Cumanayagua, Cienfuegos | Trunk-Ground | 712854 | 710491 | 710797 |
| 247 | 06_5_sag | *A. sagrei* | C7 | Cienaga de Zapata, Matanzas | Trunk-Ground | 712856 | 710492 | 710798 |
| 248 | 07_1_sag | *A. sagrei* | C8 | Cienaga de Zapata, Matanzas | Trunk-Ground | 712860 | 710494 | 710800 |
| 249 | 07_2_sag | *A. sagrei* | C12 | Manicaragua, Villa Clara | Trunk-Ground | 712861 | 710495 | 710801 |
| 250 | 07_8_sag | *A. sagrei* | W3 | Minas de Matahambre, Pinar del Río, Cuba | Trunk-Ground | 712867 | 710497 | 710803 |
| 251 | 08_4_sag | *A. sagrei* | C13 | Trinidad, Sancti Spiritus | Trunk-Ground | 712871 | 710498 | 710804 |
| 252 | 08_5_sag | *A. sagrei* | C13 | Trinidad, Sancti Spiritus | Trunk-Ground | 712872 | 710499 | 710805 |
| 253 | 08_6_sag | *A. sagrei* | C1 | Cárdenas, Matanzas | Trunk-Ground | 712873 | 710500 | 710806 |
| 254 | 25_2_sag | *A. sagrei* | E19 | Baracoa, Guantanamo | Trunk-Ground | 712875 | 710501 | 710807 |
| 255 | 26_3_sag | *A. sagrei* | E20 | Baracoa, Guantanamo | Trunk-Ground | 712877 | 710502 | 710808 |
| 256 | 26_6_sag | *A. sagrei* | E25 | Moa, Holguín | Trunk-Ground | 712880 | 710503 | 710809 |
| 257 | 26_7_sag | *A. sagrei* | E25 | Moa, Holguín | Trunk-Ground | 712881 | 710504 | 710810 |
| 258 | 27_6_sag | *A. sagrei* | E13 | San Antonio del Sur, Guantánamo | Trunk-Ground | 712883 | 710505 | 710811 |
| 259 | 27_7_sag | *A. sagrei* | E4 | Niquero, Granma | Trunk-Ground | 712884 | 710506 | 710812 |
| 260 | 28_2_sag | *A. sagrei* | E3 | Niquero, Granma | Trunk-Ground | 712887 | 710507 | 710813 |
| 261 | 28_7_sag | *A. sagrei* | E5 | Santiago de Cuba, Santiago de Cuba | Trunk-Ground | 712889 | 710508 | 710814 |
| 262 | 28_8_sag | *A. sagrei* | E5 | Santiago de Cuba, Santiago de Cuba | Trunk-Ground | 712890 | 710509 | 710815 |
| 263 | 29_7_sag | *A. sagrei* | W11 | Candelaria, Artemisa, Cuba | Trunk-Ground | 712893 | 710510 | 710816 |
| 264 | 32_7_sag | *A. sagrei* | C8 | Cienaga de Zapata, Matanzas | Trunk-Ground | 712898 | 710511 | 710817 |
| 265 | 33_5_sag | *A. sagrei* | E1 | Niquero, Granma | Trunk-Ground | 712901 | 710512 | 710818 |
| 266 | 33_7_sag | *A. sagrei* | E2 | Niquero, Granma | Trunk-Ground | 712903 | 710513 | 710819 |
| 267 | 34_4_sag | *A. sagrei* | E29 | Yateras, Guantánamo | Trunk-Ground | 712904 | 710514 | 710820 |
| 268 | 34_6_sag | *A. sagrei* | C14 | Sancti Spiritus, Sancti Spiritus | Trunk-Ground | 712906 | 710515 | 710821 |
| 269 | 34_8_sag | *A. sagrei* | C19 | Primero de Enero, Ciego de Avila | Trunk-Ground | 712908 | 710516 | 710822 |
| 270 | 36_8_all | *A. allogus* | W9 | Candelaria, Artemisa, Cuba | Trunk-Ground | 712914 | 710518 | 710824 |
| 271 | 40_6_all | *A. allogus* | E28 | Yateras, Guantánamo | Trunk-Ground | 712922 | 710521 | 710827 |
| 272 | 41_5_all | *A. allogus* | C18 | Sierra de Cubitas, Camaguey | Trunk-Ground | 712924 | 710522 | 710828 |
| 273 | 42_1_all | *A. allogus* | E19 | Baracoa, Guantanamo | Trunk-Ground | 712928 | 710523 | 710829 |
| 274 | 43_3_all | *A. allogus* | E20 | Baracoa, Guantanamo | Trunk-Ground | 712933 | 710524 | 710830 |
| 275 | 43_8_all | *A. allogus* | E21 | Baracoa, Guantanamo | Trunk-Ground | 712935 | 710525 | 710831 |
| 276 | 44_2_all | *A. allogus* | E25 | Moa, Holguín | Trunk-Ground | 712937 | 710526 | 710832 |
| 277 | 45_3_all | *A. allogus* | E25 | Moa, Holguín | Trunk-Ground | 712941 | 710528 | 710834 |
| 278 | 45_4_all | *A. allogus* | E5 | Santiago de Cuba, Santiago de Cuba | Trunk-Ground | 712942 | 710529 | 710835 |
| 279 | 45_5_all | *A. allogus* | E5 | Santiago de Cuba, Santiago de Cuba | Trunk-Ground | 712943 | 710530 | 710836 |
| 280 | 45_6_hom | *A. homolechis* | E5 | Santiago de Cuba, Santiago de Cuba | Trunk-Ground | 712944 | 710531 | 710837 |
| 281 | 48_1_hom | *A. homolechis* | W9 | Candelaria, Artemisa, Cuba | Trunk-Ground | 712951 | 710532 | 710838 |
| 282 | 49_2_hom | *A. homolechis* | C8 | Cienaga de Zapata, Matanzas | Trunk-Ground | 712956 | 710533 | 710839 |
| 283 | 49_5_hom | *A. homolechis* | E22 | Baracoa, Guantanamo | Trunk-Ground | 712957 | 710534 | 710840 |
| 284 | 50_7_hom | *A. homolechis* | E17 | Baracoa, Guantanamo | Trunk-Ground | 712961 | 710535 | 710841 |
| 285 | 50_8_hom | *A. homolechis* | E17 | Baracoa, Guantanamo | Trunk-Ground | 712962 | 710536 | 710842 |
| 286 | 52_1_hom | *A. homolechis* | E13 | San Antonio del Sur, Guantánamo | Trunk-Ground | 712963 | 710537 | 710843 |
| 287 | 52_2_hom | *A. homolechis* | E13 | San Antonio del Sur, Guantánamo | Trunk-Ground | 712964 | 710538 | 710844 |
| 288 | 53_1_hom | *A. homolechis* | E5 | Santiago de Cuba, Santiago de Cuba | Trunk-Ground | 712967 | 710540 | 710846 |
| 289 | 54_5_hom | *A. homolechis* | W11 | Candelaria, Artemisa, Cuba | Trunk-Ground | 712970 | 710541 | 710847 |
| 290 | 54_7_hom | *A. homolechis* | W11 | Candelaria, Artemisa, Cuba | Trunk-Ground | 712972 | 710542 | 710848 |
| 291 | 56_6_hom | *A. homolechis* | E27 | Yateras, Guantánamo | Trunk-Ground | 712975 | 710544 | 710850 |
| 292 | 57_1_hom | *A. homolechis* | E29 | Yateras, Guantánamo | Trunk-Ground | 712978 | 710546 | 710852 |
| 293 | 59_3_imi | *A. imias* | E36 | San Antonio del Sur, Guantánamo | Trunk-Ground | 712980 | 710547 | 710853 |
| 294 | 59_8_jub | *A. jubar* | E1 | Niquero, Granma | Trunk-Ground | 712983 | 710548 | 710854 |
| 295 | 61_3_jub | *A. jubar* | E36 | San Antonio del Sur, Guantánamo | Trunk-Ground | 712987 | 710549 | 710855 |
| 296 | 61_4_jub | *A. jubar* | E13 | San Antonio del Sur, Guantánamo | Trunk-Ground | 712988 | 710550 | 710856 |
| 297 | 61_5_jub | *A. jubar* | E13 | San Antonio del Sur, Guantánamo | Trunk-Ground | 712989 | 710551 | 710857 |
| 298 | 62_3_jub | *A. jubar* | C18 | Sierra de Cubitas, Camaguey | Trunk-Ground | 712991 | 710552 | 710858 |
| 299 | 62_6_jub | *A. jubar* | C18 | Sierra de Cubitas, Camaguey | Trunk-Ground | 712994 | 710553 | 710859 |
| 300 | 63_3_jub | *A. jubar* | C19 | Primero de Enero, Ciego de Avila | Trunk-Ground | 712995 | 710554 | 710860 |
| 301 | 64_2_con | *A. confusus* | E3 | Niquero, Granma | Trunk-Ground | 712998 | 710555 | 710861 |
| 302 | 64_8_con | *A. confusus* | E4 | Niquero, Granma | Trunk-Ground | 713000 | 710556 | 710862 |
| 303 | 66_1_rub | *A. rubribarbus* | E30 | Yateras, Guantánamo | Trunk-Ground | 713006 | 710557 | 710863 |
| 304 | 66_6_rub | *A. rubribarbus* | E17 | Baracoa, Guantanamo | Trunk-Ground | 713010 | 710558 | 710864 |
| 305 | 67_6_guaf | *A. guafe* | E1 | Niquero, Granma | Trunk-Ground | 713018 | 710559 | 710865 |
| 306 | 68_2_mes | *A. mestrei* | W9 | Candelaria, Artemisa, Cuba | Trunk-Ground | 713020 | 710560 | 710866 |
| 307 | 69_4_all | *A. allogus* | W10 | Candelaria, Artemisa, Cuba | Trunk-Ground | 713025 | 710561 | 710867 |
| 308 | 69_7_all | *A. allogus* | E28 | Yateras, Guantánamo | Trunk-Ground | 713027 | 710562 | 710868 |
| 309 | 69_8_qua | *A. quadriocelifer* | W1 | Sandino, Pinar del Río, Cuba | Trunk-Ground | 713028 | 710563 | 710869 |
| 310 | 70_2_ahl | *A. ahli* | C14 | Sancti Spiritus, Sancti Spiritus | Trunk-Ground | 713030 | 710564 | 710870 |
| 311 | 70_4_ahl | *A. ahli* | C14 | Sancti Spiritus, Sancti Spiritus | Trunk-Ground | 713032 | 710565 | 710871 |
| 312 | 70_7_ahl | *A. ahli* | C13 | Trinidad, Sancti Spiritus | Trunk-Ground | 713035 | 710566 | 710872 |
| 313 | 71_1_ahl | *A. ahli* | C13 | Trinidad, Sancti Spiritus | Trunk-Ground | 713037 | 710567 | 710873 |
| 314 | 72_3_all | *A. allogus* | W10 | Candelaria, Artemisa, Cuba | Trunk-Ground | 713039 | 710568 | 710874 |
| 315 | 73_1_sag | *A. sagrei* | E26 | Yateras, Guantánamo | Trunk-Ground | 713040 | 710569 | 710875 |
| 316 | 73_5_sag | *A. sagrei* | C14 | Sancti Spiritus, Sancti Spiritus | Trunk-Ground | 713043 | 710570 | 710876 |
| 317 | VI1_20_hom | *A. homolechis* | W5 | Viñales, Pinar del Río | Trunk-Ground | 713049 | 710573 | 710879 |
| 318 | VI1_3_mes | *A. mestrei* | W5 | Viñales, Pinar del Río | Trunk-Ground | 713051 | 710574 | 710880 |
| 319 | VI6_5_all | *A. allogus* | W7 | Viñales, Pinar del Río | Trunk-Ground | 713055 | 710576 | 710882 |
| 320 | VI9_6_bre | *A. bremeri* | W6 | Viñales, Pinar del Río | Trunk-Ground | 713057 | 710577 | 710883 |
| 321 | AY296147 | *A. porcus* | - | - | Unique Type 4 | AY296147 | - | - |
| 322 | AY296185 | *A. macilentus* | - | - | Grass-Bush | AY296185 | - | - |
| 323 | AY909766 | *A. oporinus* | - | - | Trunk-Crown | AY909766 | - | - |

**Table S1.** (b) Regional sampling; data are expressed as numbers of samples/number of localities. *: five species sequenced for the first time.

| **Species** | **Sample size**  **(# Samples / # localities)** | **Localities** | **Ecomorph** |
| --- | --- | --- | --- |
| 1. *ahli* | 4 / 2 | C (13,14) | Trunk-Ground |
| 1. *allogus West-Central* | 8 / 6 | W (3, 7, 9, 10); C (3, 18) | Trunk-Ground |
| 1. *allogus East* | 15 / 10 | E (5, 7, 10, 15, 18, 19, 20, 21, 25, 28) | Trunk-Ground |
| 1. *bremeri* | 5 / 3 | W (3, 6, 8) | Trunk-Ground |
| 1. *confusus* | 2 / 2 | E (3, 4) | Trunk-Ground |
| 1. *guafe* | 1 / 1 | E1 | Trunk-Ground |
| 1. *homolechis* | 31 / 23 | W (2, 3, 4, 5, 9, 11); C (1, 2, 3, 8, 12, 13); E (5, 7, 8, 10, 13, 17, 22, 23, 27, 29, 34) | Trunk-Ground |
| 1. *imias* | 1 / 1 | E12 | Trunk-Ground |
| 1. *jubar* | 11 / 7 | C (18, 19); E (1, 12, 13, 15. 16) | Trunk-Ground |
| 1. *mestrei* | 3 / 3 | W (3, 5, 9) | Trunk-Ground |
| 1. *rubribarbus* | 3 / 3 | E (17, 24, 30) | Trunk-Ground |
| 1. *sagrei* | 33 / 25 | W (3, 8, 11); C (1, 3, 7, 8, 12, 13, 14, 15, 17, 19); E (1, 2, 3, 4, 5, 13, 18, 19, 20, 25, 26, 29) | Trunk-Ground |
| 1. *quadriocellifer* | 4 / 2 | W (1, 2) | Trunk-Ground |
| 1. *alayoni* | 3 / 3 | E (3, 17, 19) | Twig |
| 1. *angusticeps* | 16 / 10 | W (1, 9, 10, 12); C (1, 2, 8, 16); E (1, 35) | Twig |
| 1. *guazuma* | 3 / 3 | E (5, 6, 7) | Twig |
| 1. *paternus* | 1 / 1 | W8 | Twig |
| 1. *alfaroi* | 1 / 1 | E31 | Grass-Bush |
| 1. *alutaceus* | 23 / 13 | W (1, 5, 9); C (3, 12, 13, 14, 18); E (5, 6, 7) | Grass-Bush |
| 1. *anfiloquioi** | 1 / 1 | E19 | Grass-Bush |
| 1. *clivicola* | 1 / 1 | E10 | Grass-Bush |
| 1. *cupeyalensis* | 3 / 2 | E27, E31 | Grass-Bush |
| 1. *cyanopleurus* | 7 / 4 | E (14, 15, 19, 25) | Grass-Bush |
| 1. *fugitivus** | 3 / 2 | E (24, 25) | Grass-Bush |
| 1. *inexpectatus* | 9 / 6 | E (17, 23, 24, 25, 31, 32) | Grass-Bush |
| 1. *rejectus* | 1/ 1 | E5 | Grass-Bush |
| 1. *spectrum** | 1 / 1 | C21 | Grass-Bush |
| 1. *vanidicus* | 1 / 1 | C13 | Grass-Bush |
| 1. *allisoni Central* | 4 / 3 | C (5, 6, 7) | Trunk-Crown |
| 1. *allisoni_East* | 6 / 3 | E (1, 3, 18) | Trunk-Crown |
| 1. *altitudinalis* | 1 / 1 | E10 | Trunk-Crown |
| 1. *isolepis* | 5 / 3 | E (7, 31, 35) | Trunk-Crown |
| 1. *porcatus West-Central* | 9 / 5 | W (1, 5, 9, 12); C (13) | Trunk-Crown |
| 1. *porcatus Eastern* | 5 / 4 | E (18, 20, 25, 26) | Trunk-Crown |
| 1. *equestris* | 10 / 8 | C (1, 4, 6, 10, 11, 13, 18, 20) | Crown-Giant |
| 1. *baracoae* | 1 / 1 | E19 | Crown-Giant |
| 1. *luteogularis* | 10 / 5 | W (1, 8, 9, 10, 12) | Crown-Giant |
| 1. *noblei* | 3 / 2 | E (6, 7) | Crown-Giant |
| 1. *smallwoodi* | 7 / 5 | E (8, 12, 25, 31, 33) | Crown-Giant |
| 1. *loysianus* | 4 / 3 | W (1, 10); E10 | Unique-Type 1 |
| 1. *argillaceus* | 9 / 5 | E (12, 13, 18, 20, 27) | Unique-Type 1 |
| 1. *centralis* | 1 / 1 | E35 | Unique-Type 1 |
| 1. *litoralis** | 1 / 1 | E8 | Unique-Type 1 |
| 1. *pumilus* | 10 / 6 | W (1, 8); C (2, 8, 9, 18) | Unique-Type 1 |
| 1. *ruibali** | 1 / 1 | E1 | Unique-Type 1 |
| 1. *argenteolus* | 16 / 8 | E (1, 3, 7, 11, 12, 17, 18, 20) | Unique-Type 2 |
| 1. *lucius* | 11 / 7 | W13; C (2, 12, 15, 16, 18); E (35) | Unique-Type 2 |
| 1. *bartschi* | 2 / 2 | W (5, 7) | Unique-Type 2 |
| 1. *vermiculatus* | 1 / 1 | W10 | Unique-Type 3 |
| 1. *barbatus* | 3 / 2 | W (9, 10) | Unique-Type 4 |
| 1. *chamaleonides* | 3 / 3 | C9; E (6, 10) | Unique-Type 4 |
| 1. *guamuhaya* | 1 / 1 | C13 | Unique-Type 4 |

**Table S2. GenBank sequence accession numbers of the non-Cuban specimens used in this study.**

| **No.** | **Species** | **Location** | **NAD2** |
| --- | --- | --- | --- |
| 1 | carolinensis | North America | GCA_000090745.1 |
| 4 | acutus_P_Rico | Puerto Rico | AF055926 |
| 5 | agassizi_C_America | Central America | JN112667 |
| 6 | baleatus_Hisp | Hispaniola | AY296155 |
| 7 | biporcatus_C_America | Central America | AF294286 |
| 8 | carpenteri_C_America | Central America | AY296160 |
| 9 | coelestinus_Hisp | Hispaniola | EF531472 |
| 10 | cuvieri_P_Rico | Puerto Rico | AF055973 |
| 11 | desechensis_P_Rico | Puerto Rico | EF184116 |
| 12 | dolichocephalus_Hisp | Hispaniola | AY296169 |
| 13 | fuscoauratus_C_America | Central America | AF337792 |
| 14 | gingivinus_L_Antilles | Lesser Antilles | AY909753 |
| 15 | grahami_Jama | Jamaica | AF055938 |
| 16 | gundlachi_P_Rico | Puerto Rico | AY296177 |
| 17 | haetianus_Hisp | Hispaniola | AY263042 |
| 18 | hendersoni_Hisp | Hispaniola | AY296178 |
| 19 | insolitus_Hisp | Hispaniola | AF055933 |
| 20 | krugi_P_Rico | Puerto Rico | GU057617 |
| 21 | longiceps_Hisp | Hispaniola | AY296183 |
| 22 | loveridgei_C_America | Central America | AY909759 |
| 23 | luciae_L_Antilles | Lesser Antilles | JN112697 |
| 24 | marcanoi_Hisp | Hispaniola | AY263006 |
| 25 | marron_Hisp | Hispaniola | AY296187 |
| 26 | maynardi_Cayman |  | AY902411 |
| 27 | monticola_Hisp | Hispaniola | AY296189 |
| 28 | nicefori_S_America | South America | AF337768 |
| 29 | nitens_S_America | South America | AF337807 |
| 30 | nubilis_L_Antilles | Lesser Antilles | AY909764 |
| 31 | olssoni_Hisp | Hispaniola | AF055945 |
| 32 | onca_S_America | South America | AY909765 |
| 33 | opalinus_Jama | Jamaica | AF294305 |
| 34 | ortonii_C_America | Central America | AF294288 |
| 35 | pachypus_C_America | Central America | AY909769 |
| 36 | poecilopus_C_America | Central America | AY909771 |
| 37 | polylepis_C_America | South America | AY909772 |
| 38 | princeps_S_America | South America | JN112705 |
| 39 | reconditus_Jama | Jamaica | AF294293 |
| 40 | roquet_L_Antilles | Lesser Antilles | JN112709 |
| 41 | schwartzi_LAntilles | Lesser Antilles | AY909777 |
| 42 | semilineatus_Hisp | Hispaniola | AY296201 |
| 43 | sheplani_Hisp | Hispaniola | AF055966 |
| 44 | singularis_Hisp | Hispaniola | EF531477 |
| 45 | transversalis_S_America | South America | JN112711 |
| 46 | uniformis_C_America | Central America | AY909784 |
| 47 | utiliensis_C_America | Central America | AY909785 |
| 48 | websteri_Hisp | Hispaniola | AY296205 |
| 49 | zeus_C_America | Central America | AY909786 |

**Table S3.** Description of Unique Type in Cuba Anoles.

| Type | Species | Description |
| --- | --- | --- |
| Unique-type 1 | *A. argillaceus,*  *A. pumilu*  *A. loysianu* | *A. argillaceus* and *A. pumilu* are typically found on tree trunks, but often occur on narrow twigs in bushes no more than 2 m tall. They possess short hind limbs, similar to those of twig anoles, but their forelegs tend to be longer. These species are usually found in open habitats. By contrast, *A. loysianus* has previously been regarded as the only Cuban member of the trunk ecomorph . Nevertheless, it is considered as a unique-type 1 anole here because its morphology is not remarkably different from *A. argillaceus* and *A. pumilus*, and our fieldwork experience suggests that these three species share ecological characteristics, such as the kind of structural habitat*.* |
| Unique-type 2 | *A. argenteolus*  *A. Lucius*  *A. bartschi* | *Anolis argenteolus* and *A. lucius* show similar habitat use (rock walls and tree trunks), although *A. argenteolus* uses a wider range of trees and is less likely to be found on rock walls than *A. lucius*. *Anolis bartschi* resides almost exclusively on cliffs and other large vertical rock surfaces. |
| Unique-type 3 | *A. vermiculatus* | This species is found exclusively in the vicinity of streams. It takes refuge in water to escape from predators either by diving into the water or by running bipedally across the water. As only one locality was available for this species, it was omitted from the analysis of intraspecific genetic distances; however, it was considered in the divergence time estimation. |
| Unique-type 4 | *A. barbatus*  *A. chamaeleonides* | These anole species are large in size with relatively short limbs and tails, and have previously been considered giant twig anoles due to their morphological and ecological similarities with members of the twig ecomorph. They usually move very slowly, and adults are typically found in shaded situations and perching high on big trees. The species in this category were also omitted from the analyses of factors affecting intraspecific genetic distances because only one species (*A. chamaleonides*) with sufficient numbers of sampling localities was available; however, they were considered in the divergence time estimation. |

**Table S4.** Environmental variables for each locality. PC1 to PC5 values indicate principle component axis using 19 environmental variables from WorldClim data base and percent tree cover from MODIS s variable from sensor of Terra from International Steering Committee for Global Mapping. 19 environmental variables were Annual Mean Temperature, Mean Diurnal Range, Isothermality, Temperature Seasonality,Max Temperature of Warmest Month, Min Temperature of Coldest Month, Temperature Annual Range (BIO5-BIO6), Mean Temperature of Wettest Quarter, Mean Temperature of Driest Quarter, Mean Temperature of Warmest Quarter, Mean Temperature of Coldest Quarter, Annual Precipitation, Precipitation of Wettest Month, Precipitation of Driest Month, Precipitation Seasonality (Coefficient of Variation), Precipitation of Wettest Quarter, Precipitation of Driest Quarter, Precipitation of Warmest Quarter, Precipitation of Coldest Quarter.

| Site | PC1 | PC2 | PC3 | PC4 | PC5 |
| --- | --- | --- | --- | --- | --- |
| W2 | 0.314921849 | -1.553473752 | -0.242021339 | -0.711556316 | 2.064583837 |
| W1 | 0.578235302 | -1.697080562 | -0.509692207 | -1.015061899 | 0.945979127 |
| W8 | 1.872561562 | -1.575300734 | -1.349048145 | -0.985380886 | -0.31500212 |
| W3 | 0.61330943 | 0.78553336 | -2.359648384 | -0.804854651 | 0.089485478 |
| W4 | 0.270307303 | -0.711118828 | -2.366049467 | -0.536253326 | 0.322709409 |
| W6 | 0.548351512 | 0.302275504 | -2.476224358 | -0.85123901 | -0.215875031 |
| W15 | 0.351994566 | 0.73133283 | -2.542319481 | -0.779070592 | -0.008239121 |
| W7 | 0.654114473 | 0.382248877 | -1.96994342 | -0.493697417 | -0.610932952 |
| W5 | 0.892250593 | 0.213875035 | -2.000934148 | -0.693310672 | -1.246651386 |
| W9 | 0.590287692 | 1.133452552 | -2.095522274 | -0.484010843 | -0.537621008 |
| W10 | 0.03029281 | 1.965869119 | -2.246373602 | -0.303201085 | 0.454024536 |
| W11 | 0.2138544 | 0.951791541 | -1.884474454 | -0.2736073 | 0.504506117 |
| W12 | 1.172451274 | -1.564134293 | -0.40168244 | -3.115913527 | -0.695838355 |
| C4 | 1.439224213 | -1.768275148 | 0.036336484 | -2.072918559 | -0.740670966 |
| W13 | 2.162627118 | 0.349013007 | -1.911776589 | 0.433301303 | -1.316207951 |
| C3 | 1.39284693 | 0.690324426 | -1.656894015 | -0.466281314 | -0.770783439 |
| C2 | 0.944580543 | -1.373409572 | -0.574433975 | -0.933811685 | 0.863819177 |
| C7 | 3.563050451 | -0.561936532 | -0.708000218 | 2.282068553 | 1.173129058 |
| C1 | 2.276889912 | -2.041489594 | -1.197465932 | -1.429723124 | 0.201608862 |
| C11 | 3.386559239 | -0.549924288 | -0.135758806 | 1.500880852 | 1.426895239 |
| C8 | 3.386559239 | -0.549924288 | -0.135758806 | 1.500880852 | 1.426895239 |
| C5 | 4.056114893 | -0.689443302 | -1.184979278 | 2.444101369 | -1.075955622 |
| C6 | 4.064623252 | -0.600247621 | -1.481488625 | 2.653096387 | -1.681623664 |
| C10 | 3.297122838 | -0.756484617 | 0.205372854 | 0.637969024 | 1.408110577 |
| C9 | 2.713884024 | -1.076828521 | -1.535000294 | 1.481903358 | -0.963678622 |
| C15 | 2.091590197 | -0.550040043 | 0.336542437 | 0.476113507 | 1.213755217 |
| C12 | 1.078584674 | 1.231007483 | -1.708042509 | 0.119847203 | 0.595240095 |
| C17 | 2.132708075 | -1.632604726 | -0.176878947 | -0.160974224 | 1.055368348 |
| C13 | -0.729973483 | 4.756298421 | -2.809831054 | -0.940284912 | 1.321329501 |
| C16 | 2.376416471 | -1.909439164 | -0.153805537 | -0.306733921 | 0.208799371 |
| C14 | 0.186287847 | 3.812112878 | -2.51257611 | -0.501812582 | 1.177737898 |
| C20 | 1.805753922 | -0.140054721 | -2.311825385 | -1.763864968 | -0.887400509 |
| C19 | 3.485682757 | -1.964870174 | -1.110917647 | 0.684615625 | -0.992229245 |
| C18 | 2.358919078 | -0.700207151 | -0.303996088 | 0.487743 | -0.525902011 |
| E1 | 1.596398049 | -2.80245707 | 3.99523537 | -0.045616124 | 1.260182577 |
| E4 | 1.600528971 | -2.540509707 | 3.806596657 | 0.136451935 | 1.482576335 |
| E2 | 2.270474422 | -2.834281522 | 3.325217577 | 0.043720522 | -0.689215013 |
| E3 | 1.000193438 | -0.229850787 | 2.949139296 | 0.280998127 | 0.712887763 |
| E11 | 1.021041451 | 0.790494837 | 2.575351157 | 0.801167622 | -0.251186927 |
| E6 | -0.548791846 | 4.729960016 | 0.812060308 | 0.834477163 | 0.03916475 |
| E10 | -0.548791846 | 4.729960016 | 0.812060308 | 0.834477163 | 0.03916475 |
| E7 | 1.07402134 | 1.098943349 | 0.956061786 | 1.546830771 | -0.565539616 |
| E35 | 1.395945169 | -4.129247759 | 2.886787179 | -2.675146615 | 0.056515899 |
| E5 | -1.214511718 | 5.802994296 | 1.062859493 | -0.682084102 | 0.774157223 |
| E8 | 1.375728532 | 0.061183304 | 2.026591934 | 0.38995973 | -0.221117562 |
| E34 | 1.375327655 | -0.694808029 | 2.006919902 | 0.238629358 | -1.056285453 |
| E31 | -1.490408886 | 5.83391955 | 1.258792728 | -0.677279854 | -0.703681477 |
| E12 | 0.769577198 | -1.234469176 | 3.285808215 | -0.303785324 | -0.941374587 |
| E26-28 | -1.104575156 | 3.08572069 | 1.658769506 | 0.601677287 | -0.838343272 |
| E29 | -1.612878571 | 4.337607783 | 1.62813454 | 0.393698752 | -0.188192018 |
| E30 | -1.612878571 | 4.337607783 | 1.62813454 | 0.393698752 | -0.188192018 |
| E36 | 0.769577198 | -1.234469176 | 3.285808215 | -0.303785324 | -0.941374587 |
| E33 | -2.276348462 | 4.542799295 | 1.271641239 | -0.072877459 | 0.023296996 |
| E25 | -2.276348462 | 4.542799295 | 1.271641239 | -0.072877459 | 0.023296996 |
| E13 | -1.266607415 | -1.130356745 | 2.276268608 | -0.344156057 | -1.333316183 |
| E24 | -3.726008907 | 0.92819578 | 0.535253471 | 0.114109849 | -0.241465619 |
| E23 | -4.919471178 | -2.610772354 | -0.263269686 | 0.482895155 | -0.071466299 |
| E32 | -5.336520609 | -2.97229028 | -0.578090193 | 0.598572458 | 0.005104817 |
| E17 | -5.336520609 | -2.97229028 | -0.578090193 | 0.598572458 | 0.005104817 |
| E22 | -5.78003153 | -2.559471671 | -0.924694338 | 0.845101718 | -0.244314287 |
| E21 | -6.531960207 | -3.399040829 | -1.548289247 | 0.729010667 | -0.153161913 |
| E20 | -6.690225542 | -2.173342147 | -1.553004755 | 1.046730177 | -0.496573301 |
| E14 | -2.669471696 | 1.075053665 | 2.208347485 | -0.535574619 | -0.273140982 |
| E19 | -5.341914573 | -2.680836421 | -0.134150027 | 0.126444456 | -0.61915911 |
| E18 | -7.150480974 | -3.589937077 | -1.252258886 | 0.965368898 | 1.675954526 |
| E15 | -1.898245038 | 2.06782745 | 3.096491213 | -1.147468687 | 0.0592177 |

**Table S5**. Results of a phylogenetic generalized least squares (PGLS) analysis for the final model using both nuclear DNA (nDNA) and mitochondrial DNA (mtDNA) (a) and only mtDNA (b). The effects of species age, ecomorph, and environmental heterogeneity within a species’ range on the average genetic divergence among populations of a species was examined. Instead of geographic distance, species ragen was included as a covariate that potentially affects genetic divergence. The PGLS analysis was performed using a full model that included all of the explanatory variables and possible interaction terms. Non-significant variables were then removed from the full model with a backward stepwise procedure using log-likelihood ratio tests. The final model that included only significant variables and their interaction terms was then analyzed.

| (a) nDNA + mtDNA | | | | | | (b) mtDNA | | | | |
| --- | --- | --- | --- | --- | --- | --- | --- | --- | --- | --- |
|  | d.f. | F-value | *P*-value |  |  |  | d.f. | F-value | *P*-value |  |
| (Intercept) | 1 | 4.883688 | **0.0628** |  |  |  | 1 | 8.491575 | **0.0195** |  |
| Age | 1 | 14.405765 | **0.0068** |  |  | Age | 1 | 4.789919 | **0.0049** |  |
| Range | 1 | 10.518995 | **0.0142** |  |  | Range | 1 | 26.923957 | **0.0008** |  |
| Environmental heterogeneity | 1 | 0.052158 | 0.8259 |  |  | Environmental heterogeneity | 1 | 0.904156 | 0.3695 |  |
| Ecomorph | 6 | 1.041304 | 0.4721 |  |  | Ecomorph | 6 | 0.940905 | 0.5159 |  |
| Range × Age | 1 | 0.995732 | 0.3516 |  |  | Range × Age | 1 | 2.139683 | 0.1817 |  |
| Range × environmental heterogeneity | 1 | 1.498789 | 0.2605 |  |  | Age × environmental heterogeneity | 1 | 0.838170 | 0.3867 |  |
| Range × ecomorph | 6 | 2.418368 | 0.1365 |  |  | Range × ecomorph | 6 | 6  3.177635 | 0.0670 |  |
| Age × environmental heterogeneity |  | 2.366212 | 0.1679 |  |  |  |  |  |  |  |
