# Supplementary material for: Factors affecting interspecific differences in genetic divergence among populations of Anolis lizards in Cuba
Source: Zoological Lett. 2018 Aug 9;4:21. doi: 10.1186/s40851-018-0107-x (PMC6085692; doi:10.1186/s40851-018-0107-x)
Supplement: Supplementary file 2 — Figure S1. Bayesian tree (50% majority consensus) based on three genes using 303 individuals from 33 Cuban Anolis species and two species from the genus Leiocephalus. Figure S2. Contrasting Bayesian phylogenies for nuclear DNA (based on ZNF521 and FBRSL1) and mtDNA (based on ND2) for Cuban anoles. Figure S3. Phylogenetic tree with estimates of divergence times based on the mitochondrial gene ND2 (1036 bp). (DOC 8930 kb) [file 40851_2018_107_MOESM2_ESM.doc]

**Figure S1** Bayesian tree (50% majority consensus) based on three genes (2170 bp); 303 individuals from 33 Cuban *Anolis* species were included as the ingroup and two species from the genus *Leiocephalus* were used as the outgroup. Node supports are Bayesian posterior probabilities (*P* > 0.95) and bootstrap percentages from maximum likelihood analyses (*P* > 90%), and are shown only for major clades. The figure has been split into parts A, B, and C, and some clades have been compressed to fit the full tree. CG, crown-giant; GB, grass-bush; T, twig; TC, trunk-crown; TG, trunk-ground; U1, unique-type 1; U2, unique-type 2; U4, unique-type 4; outgroup not shown

**Figure S2.** Contrasting Bayesian phylogenies for 1134 bp of nuclear DNA (based on ZNF521 and FBRSL1) and 1036 bp of mtDNA (based on ND2) for Cuban anoles (50% majority-rule consensus). Branches indicated with thin and dotted lines are low Bayesian posterior probabilities (0.98–0.95, and 0.94–0.50, respectively).

**Figure S3** Phylogenetic tree with estimates of divergence times based on the mitochondrial gene *ND2* (1036 bp); 224 individuals from 54 Cuban *Anolis* species and 47 non-Cuban species were included. Node ages and 95% confidence intervals (CI) are shown only for the major clades of species from Cuba. The figure has been split into parts A, B, and C and some clades have been compressed to fit the full tree. CG, crown-giant; GB, grass-bush; T, twig; TC, trunk-crown; TG, trunk-ground; U1, unique-type 1; U2, unique-type 2; U4, unique-type 4; outgroup not shown
